# The Protective Effect of Rosmarinic Acid against Unfavorable Influence of Methylparaben and Propylparaben on Collagen in Human Skin Fibroblasts

**DOI:** 10.3390/nu12051282

**Published:** 2020-05-01

**Authors:** Natalia Matwiejczuk, Anna Galicka, Ilona Zaręba, Małgorzata M. Brzóska

**Affiliations:** 1 Department of Medical Chemistry, Medical University of Bialystok, Adama Mickiewicza 2A street, 15-222 Bialystok, Poland; 2 Department of Medicinal Chemistry, Medical University of Bialystok, Adama Mickiewicza 2D street, 15-222 Bialystok, Poland; ilona.zareba@gmail.com; 3 Department of Toxicology, Medical University of Bialystok, Adama Mickiewicza 2C street, 15-222 Bialystok, Poland; malgorzata.brzoska@umb.edu.pl

**Keywords:** rosmarinic acid, methylparaben, propylparaben, collagen, ERK1/2, MMP, TIMP, proliferation, apoptosis, skin fibroblasts

## Abstract

Parabens, which are widely used in food, medicines and cosmetics, have a harmful effect on human health. People are most exposed to parabens transdermally by using cosmetic products containing these preservatives. The purpose of this study was to estimate the influence of parabens (methylparaben—MP and propylparaben—PP) on the metabolism of collagen in the human skin fibroblasts and above all, to assess whether rosmarinic acid (RA—50, 100, or 150 μM) can protect these cells from the adverse effects of parabens (0.001% MP and 0.0003% PP, 0.003% MP and 0.001% PP, and 0.01% MP and 0.003% PP). The possible mechanisms of RA action were estimated as well. Parabens decreased the expression of collagen type I and III at mRNA and protein levels, while RA (depending on the concentration) provided partial or total protection against these changes. The effective protection against the adverse effects of parabens on cell viability and proliferation was also provided by RA. The beneficial impact of RA on collagen and the fibroblasts resulted from an independent action of this compound and its interaction with parabens. This study allows us to conclude that this polyphenolic compound may protect from unfavorable health outcomes caused by lifetime human exposure to parabens contained in cosmetic products.

## 1. Introduction

**Figure 1:**
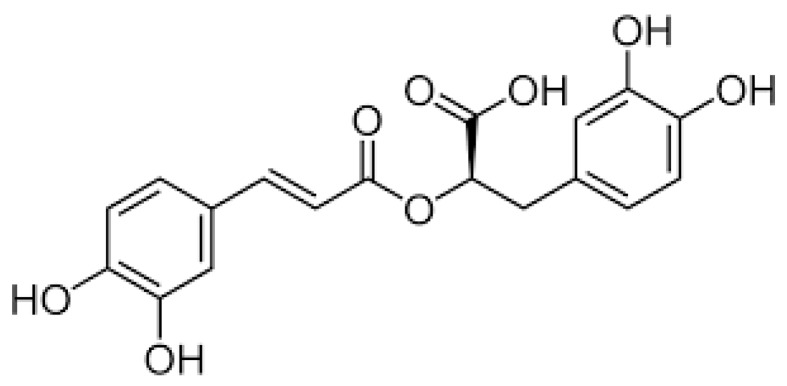
Chemical structure of rosmarinic acid (RA).

Rosmarinic acid (RA) is an ester of caffeic acid and 3,4-dihydroxy-phenyllactic acid (α-o-caffeoyl-3,4-dihydroxyphenyl lactic acid) (Figure 1). It is a polyphenolic compound occurring in many species of herbs including rosemary (*Rosmarinus officinalis* L.), perilla (*Perilla frutescens* L.), basil (*Ocimum basilicum* L.), oregano (*Origanum vulgare* L.), lemon balm (*Melissa officinalis* L.), marjoram (*Origanum majorana* L.), sage (*Salvia officinalis* L.), and thyme (*Thymus vulgaris* L.) [1,2]. These herbs have long been widely used in traditional medicine in states of exhaustion and weakness of the body, in inflammation, infection, and depression, to improve memory, in hepatic, renal, and cardiovascular diseases, as well as for indigestion and gastritis [3,4]. Their therapeutic effects have been shown to be associated mainly with RA, which exhibits a wide range of valuable biological activities such as antimicrobial, antiviral, antioxidant, anti-inflammatory, antiangiogenic, anti-depressant, antihyperglycemic, anti-allergic, antithrombotic, anticarcinogenic, and anti-aging [3,4,5,6,7]. In addition, all these herbs and their extracts containing RA have been used as food additives to improve not only the taste and smell, but also to increase the nutritional value. Moreover, RA, due to its antimicrobial and antioxidant properties, is used to prevent oxidation and growth of microorganisms, which extends food durability [4,5,8]. Therefore, it has potential to replace or at least reduce, the amount of synthetic antioxidants and preservatives commonly used not only in the food industry, but also in the pharmaceutical and cosmetics industries.

The strong antioxidant and anti-inflammatory properties of RA and containing these acid extracts have also been used to protect the skin against the unfavorable impact of many external factors such as ultraviolet (UV) radiation and reactive oxygen species (ROS) [9,10,11]. The skin is the largest organ of the human body, which supports homeostasis and serves as the protective shield against sunlight, heat, infections or absorption of chemical substances [12]. Collagen, produced by the skin fibroblasts, is the major protein involved in the construction of the human skin, and is responsible for elasticity and stiffness of the skin, as well as its tensile strength [13,14]. In addition, collagen plays an important role in retaining the moisture in the skin, which is extremely important in the maintaining of healthy and good-looking skin [15,16]. Nowadays, a very popular and important aspect of life is skin care (as well as embellishing) with the help of many different cosmetic products. The regular use of these cosmetics can protect the skin against external factors, improve skin elasticity and smoothness, accelerate its regeneration and delay the aging processes [17,18,19]. Cosmetic formulations contain in their composition, among others, vitamins and polyphenolic compounds, which by decreasing the concentration of free radicals can exert a beneficial effect on the skin health. However, all these cosmetic products, except for the bioactive ingredients, contain a lot of chemical substances with adverse effects on the skin, among them synthetic preservatives including parabens.

Parabens are esters of *p*-hydroxybenzoic acid (PHBA) and the most commonly used in cosmetics are methylparaben (MP), ethylparaben (EP), n-propylparaben (PP), and n-butylparaben (BP) [20,21]. Although the acceptable concentrations: 0.4% for MP and EP, 0.14% for PP and BP, and 0.8% for the mixture of these parabens were considered safe [22,23], there are concerns regarding the lifetime exposure of human to these compounds, mainly due to their ability to interfere with estrogen metabolism, demonstrated in many in vitro and in vivo studies [20,24,25,26] and recently summarized by us [27]. The widespread use of parabens in cosmetics may be associated with a higher risk of development of breast cancer, obesity, gestational diabetes, malignant melanoma, and reproductive disorders (for a review, see [27]). In addition, exposure to these compounds via the skin may also cause allergic symptoms [25,27].

Our research team previously provided data on the harmful effects of MP alone at concentrations of 0.01%, 0.03%, and 0.05% on the expression of collagen in the human skin fibroblasts [28]. In the present study, to better reflect the real situation of human exposure to parabens, we have used the lowest concentration of MP (0.01%), at which we have noted its unfavorable impact on the skin cells, and two lower concentrations (0.001% and 0.003%). They were within the same range as in most of the studies on parabens performed on the human fibroblasts and keratinocytes (summarized recently by us; for a review, see [27]). The lower concentrations of MP (0.001% and 0.003%) are considered to be practical and they are based on the assumption that the typical concentrations of MP in cosmetics are about 0.32% and below [27,29], and about (0.025%–1%) reaches the epidermis [30,31,32]. Because most often, more than one paraben is used to increase the effectiveness of preservatives, we have decided to investigate the influence of a mixture of MP with PP on this main skin protein. These two parabens are among the most commonly used in cosmetics and the most frequently, and in the highest concentration detected in the human body [20,21,24,25,27]. Since the most common preservative system in cosmetic formulations contains 0.3% MP and 0.1% PP [20,33] (Table S1), parabens in the same ratio (3:1) were also used in our study to more realistically reflect the exposure of human to parabens via cosmetics. However, the most important assumption of this study was that RA, which has multidirectional biological activity, can counteract the negative changes caused by parabens in the skin cells. In addition, attempts to search for possible mechanisms of the protective action of RA against the unfavorable effect of these preservatives on the metabolism of collagen in the skin fibroblasts were made. Therefore, the expression of factors involved in the regulation of synthesis, secretion and degradation of collagen such as extracellular signal-regulated protein kinases 1 and 2 (ERK1/2), collagen triple helix repeat containing-1 (CTHRC1), heat shock protein, 47 kDa (HSP47), matrix metalloproteinases (MMP-1, MMP-2), membrane type-1 matrix metalloproteinase (MT1-MMP), and tissue inhibitors of matrix metalloproteinases (TIMP-1, TIMP-2) was estimated. In addition, expression of some apoptotic (BCL2-associated X protein—Bax, cleaved caspase-3) and antiapoptotic (B-cell lymphoma-extra large antiapoptotic protein—Bcl-xL) markers was also evaluated. This study reveals new clinically relevant properties of RA associated with the potential of this polyphenolic compound to counteract the unfavorable effects of MP and PP on the collagen metabolism in the human skin fibroblasts.

## 2. Materials and Methods 

### 2.1. Reagents 

MP, PP, radioimmunoprecipitation assay (RIPA) buffer, [3-(4,5-dimethylthiazol-2-yl)-2,5- diphenyltetrazolium bromide] (MTT), sodium dodecyl sulfate (SDS), dimethyl sulfoxide (DMSO), gelatin, bovine serum albumin (BSA), acrylamide and N,N’-Methylenebisacrylamide, bacterial collagenase, and protease inhibitor cocktail (P8340), and N-ethylmaleimide (NEM) were provided by Sigma-Aldrich Corp. (St. Louis, MO, USA). Phosphatase inhibitor cocktail (524625-1SET) was purchased from Merck Millipore Ltd. (Carrigtwohill, County Cork, Ireland). RA was a product of BIOKOM (Warsaw, Poland). Dulbecco’s minimal essential medium (DMEM), phosphate-buffered saline (PBS), and fetal bovine serum (FBS) used in the cell culture were purchased from Gibco (Thermo Fisher Scientific, Waltham, MA, USA). Penicillin, streptomycin, and glutamine were obtained from Quality Biologicals Inc. (Gaithersburg, MD, USA). [^3^H]thymidine (6.7 Ci/mmol) and L-5[^3^H] proline (28 Ci/mmol) were purchased from Amersham Biosciences (Buckinghamshire, UK).

### 2.2. Cell Culture

Normal human skin fibroblasts (CRL-1474) were purchased from American Type Culture Collection (Manassas, VA, USA). The cells were grown in the culture medium DMEM supplemented with 10% FBS, 2 mM glutamine, 50 U/mL penicillin, and 50 μg/mL streptomycin at 37 °C in a 5% CO_2_ incubator. For the experiments, the culture medium was replaced with the serum-free medium. MP, PP, and RA were dissolved in 99.5% ethanol and stored as the concentrated solutions at 4 °C. For the experiments, they were appropriately diluted in DMEM and added to the cells in such volumes that the final concentration of ethanol did not exceed 0.1%. In each experiment, fibroblasts were incubated for 24 h in the serum-free culture medium in the presence of parabens (MP and PP) alone, parabens combined with RA, and RA alone, and an appropriate amount of the solvent (ethanol), and the latter constituted the controls. The tested compounds were used at the following concentrations: parabens (0.001% MP and 0.0003% PP, 0.003% MP and 0.001% PP, and 0.01% MP and 0.003% PP), and RA: 50, 100, 150 µM.

### 2.3. MTT Assay for Assessment of Cytotoxicity

The skin cells (1 × 10^5^ cells/well) were cultured on 24-well plates to obtain 70% of confluency and then incubated with the studied compounds for 24 h at 37 °C. After this time, the medium was removed, and cells were washed with PBS. Then, 1 mL of MTT solution (0.5 mg/mL) was added to each well and incubation was prolonged for 4 h at 37 °C. Then, the medium containing MTT solution was replaced with 1 mL of 0.1 M hydrochloric acid (HCl) dissolved in absolute isopropanol and shaken thoroughly to solubilize the formazan crystals. The absorbance was measured at 570 nm using an Asys UVN 340 microplate reader (Biogenet, Józefów, Poland). The results were expressed as the percent of viable treated cells compared to the control cells.

### 2.4. Cell Proliferation Assay

The proliferation of cells was measured using test of incorporation of [^3^H]thymidine into deoxyribonucleic acid (DNA). Cells were plated in 24-well culture dishes at 1 × 10^5^ cells/well in 1 mL of DMEM. After 48 h, the cells were incubated with various concentrations of the studied compounds (MP, PP, RA) and [^3^H]thymidine (0.5 μCi, 6.7 Ci/mmol) for 24 h at 37 °C. After this time, the medium was aspirated, and the cells were rinsed three times with PBS and then, solubilized in 1 mL of 0.1 M sodium hydroxide (NaOH) solution containing 1% SDS. The radioactivity of [^3^H]thymidine incorporated into DNA was counted in Liquid Scintillation Analyzer Tri-Carb 2810 TR (PerkinElmer, Waltham, MA, USA) after adding to each sample 2 mL of scintillation liquid (Ultima Gold XR, PerkinElmer, Waltham, MA, USA).

### 2.5. Determination of Collagen Biosynthesis 

Collagen biosynthesis in fibroblasts was determined by the test of incorporation of the radioactive L-[^5^H]proline into collagen according to the method of Peterkofsky et al. [34]. Fibroblasts were grown in DMEM containing 10% FBS in six-well plates until confluence. Then, the medium was replaced with serum-free medium for 1 h and then, the tested compounds (MP, PP, RA) at the appropriate concentrations, as well as L- [^5^H]proline (5 μCi, 28 Ci/mM) were added, after which the incubation lasted 24 h. The cells were washed twice with PBS containing 10 mM proline, then resuspended in the same solution (1.5 mL) and ultrasonically homogenized (3 × 20 s at 0 °C). To precipitate proteins, equal volumes of a 20% trichloroacetic acid (TCA) solution containing 20 mM proline were added. After that, centrifugation at 1000 × g at 4 °C for 10 min was performed and the pellets were dissolved in 0.6 mL of 0.2 M NaOH. Lysates were divided into two samples (test and control) with a volume of 0.2 mL and neutralized by adding 0.16 mL of 0.15 M HCl and 0.1 mL of 1 M Tris-HCl, pH 7.2. The solution containing 20 μL of 62.5 mM NEM, 10 μL of 25 mM calcium chloride (CaCl_2_), and 10 μL of PBS was added to both tested and control samples, whereas bacterial collagenase (C0773, Sigma-Aldrich) (1 mg per mL) was added only to test tubes, and those without collagenase constituted the controls. All samples were incubated for 90 min at 37 °C and after that, cold 10% TCA (0.5 mL) was added and then, samples were cooled for 5 min at 0 °C. Finally, the samples were centrifuged, and supernatants were transferred to scintillation vials containing 4 mL of scintillation liquid (PerkinElmer, Waltham, MA, USA). The radioactivity was counted in Liquid Scintillation Analyzer Tri-Carb 2810 TR (PerkinElmer, Waltham, MA, USA). Biosynthesis of collagen was expressed in dpm of [^3^H]proline incorporated into proteins susceptible to bacterial collagenase per mg of protein. Concentration of protein was measured using the BCA Protein Assay Kit (Pierce, Rockford, IL, USA).

### 2.6. Analysis of Activity of MMP-1 and MMP-2 by Zymography 

For analysis of an activity of MMP in the serum-free conditioned media, sodium dodecyl sulfate-polyacrylamide gel electrophoresis (SDS-PAGE) was used [35]. Equal amounts of proteins (10 μg) were mixed with non-reducing 5x Laemmli sample buffer [35] and loaded without boiling on 10% polyacrylamide gel containing 0.1% SDS and 1 mg/mL of type I collagen (Sigma-Aldrich Corp., St. Louis, MO, USA) or gelatin (Sigma-Aldrich Corp., St. Louis, MO, USA) for detection of MMP-1 and MMP-2, respectively. After electrophoresis, the gels were soaked in 2.5% Triton X-100 for 30 min at room temperature (RT) to remove SDS. After that, they were incubated overnight in 50 mM Tris-HCl, pH 8.0 buffer containing 5 mM CaCl_2_, 5 μM zinc chloride (ZnCl_2_) and 0.02% sodium azide (NaN_3_) at 37 °C to allow MMP digestion of their substrates. Gels were stained with 0.5% Coomassie Brilliant Blue R-250 in 40% methanol and 10% acetic acid until clear bands of lysis appeared on the blue background of stained gelatin or collagen that corresponded to MMP-2 and MMP-1 activities. The density of these bands were quantified using an imaging densitometer (G:BOX, Syngene, Cambridge, UK). 

### 2.7. Western Immunoblotting 

Cellular proteins were extracted at 4 °C using RIPA lysis buffer supplemented with protease inhibitor cocktail and phosphatase inhibitor cocktail in the case of testing kinase activity. The same amount of proteins (20 or 30 μg) of the cellular extracts or concentrated (Centrifugal Filter Units (10K)) (Merck Millipore Ltd., Carrigtwohill, County Cork, Ireland) 10-times conditioned media were separated on 7.5%–12% (depending on the molecular weight of the analyzed proteins) polyacrylamide gels under reducing conditions and electrotransferred onto Immobilon-P Transfer membranes (Merck Millipore Ltd., Carrigtwohill, County Cork, Ireland). The membranes were blocked with 5% non-fat milk in Tris-buffered saline (50 mM Tris-HCl, pH 7.5, 150 mM NaCl) containing 0.05% Tween 20 (TBS-T) for 1 h at RT and then rinsed three times with TBS-T. Subsequently, they were incubated overnight at 4 °C with: monoclonal antibodies against collagen I (COL1A1), collagen III (COL3A1), total ERK1/ERK2, HSP47, CTHRC1, MMP-1, and MT1-MMP (Santa Cruz Biotechnology Inc., Santa Cruz, CA, USA) at a dilution of 1:1000; with monoclonal antibody against Bax (Santa Cruz Biotechnology Inc., Santa Cruz, CA, USA) at a dilution of 1:300; monoclonal antibodies against Bcl-xL and MMP-2 (Santa Cruz Biotechnology Inc., Santa Cruz, CA, USA) at a dilution of 1:500; monoclonal antibodies against TIMP-1 and TIMP-2 (Santa Cruz Biotechnology Inc., Santa Cruz, CA, USA) at a dilution of 1:700; monoclonal antibodies against caspase-3 and phosphorylated ERK1/ERK2 (Cell Signaling Inc., Danvers, MA, USA) at a dilution of 1:1000; and monoclonal antibody against β-actin (Sigma-Aldrich Corp., St. Louis, MO, USA) at a dilution of 1:1000. All of these dilutions were performed using TBS-T containing 3% BSA. In order to analyze collagen I and III, ERK1/ERK2, HSP47, CTHRC1, Bax, Bcl-xL, caspase-3, MMP-1, MMP-2, MT1-MMP, TIMP-1, and TIMP-2, a secondary antibody, peroxidase-conjugated anti-mouse immunoglobulin G (IgG) (whole molecule) (Sigma-Aldrich Corp., St. Louis, MO, USA) was added. In order to analyze phosphorylated ERK1/ERK2 and β-actin, a secondary antibody, peroxidase-conjugated anti-rabbit IgG (whole molecule) (Cell Signaling Inc., Danvers, MA, USA) was added. Both secondary antibodies were added at the concentration of 1:2000 in TBS-T containing 5% dried milk and the membranes were incubated for 60 min under gentle shaking. Then, they were rinsed with TBS-T (5 times for 5 min) and subjected to Westar Supernova Chemiluminescent Substrate for Western Blotting (Cyanagen, Bologna, Italy). In some experiments, membranes were stripped using a Restore Western Blot Stripping Buffer (Thermo Fisher Scientific, Waltham, MA, USA) and reprobed. Western blots were carried out in at least three independent experiments. Photos of membranes were taken using apparatus for gel documentation BioSpectrum Imaging System (UVP, Upland, CA, USA). The intensity of the bands were measured by densitometry using an imaging densitometer (G:BOX, Syngene, Cambridge, UK). The signals of each band of tested proteins were normalized to the corresponding β-actin as a loading control.

### 2.8. Quantitative Real-Time Polymerase Chain Reaction (PCR)

Total ribonucleic acid (RNA) was isolated using a Total RNA Mini Plus Concentrator (A&A Biotechnology, Gdynia, Poland) according to the manufacturer’s instructions. Concentration of isolated RNA was measured with the use of a NanoDrop 2000 spectrophotometer (Thermo Fisher Scientific, Waltham, MA, USA). Equal amounts (1 μg) of total RNA were reverse transcribed using a complementary deoxyribonucleic acid (cDNA) Synthesis Kit (Bioline, London, UK) in a final volume of 20 μL. The reaction was incubated for 10 min at 45 °C, 30 min at 45 °C, and finally at 70 °C for 5 min. Quantitative real-time PCR was performed in the thermocycler CFX96 Real-Time System (Bio-Rad, Hercules, CA, USA) using a SensiFAST™ SYBR Kit (Bioline, London, UK). The reaction mixture contained 5 μL of 2 × SensiFAST SYBR No-ROX Mix, 2 μL of 3-times diluted cDNA template, 0.4 μL of each target-specific primer (Genomed, Warsow, Poland) at the concentration of 10 µM and nuclease-free water to a final volume of 10 μL. The following primers (forward and reverse) were used: *COL1A1*, forward 5’-TAC AGC GTC ACT GTC GAT GGC-3’ and reverse 5’-TCA ATC ACT GTC TTG CCC CAG-3’; *COL1A2*, forward 5’-CAC CCA GAG TGG AGC AGT GG-3’ and reverse 5’-TTC TTG GCT GGG ATG TTT TCA-3’; C*OL3A1*, forward 5’-AAT TTG GTG TGG ACG TTG GC-3’ and reverse 5’-TTG TCG GTC ACT TGC ACT GG-3’; *CTHRC1*, forward 5’-TGG ACA CCC AAC TAC AAG CA-3’ and reverse 5’-GAA CAA GTG CCA ACC CAG AT-3’; *HSP47*, forward 5’-AAC TGC GAG CAC TCC AAG A-3’ and reverse 5’-ATG AAG CCA CGG TTG TCC-3’; *MMP-1*, forward 5’-ATT GGA GCA GCA AGA GGC TGG GA-3’ and reverse 5’-TTC CAG GTA TTT CTG GAC TAA GT-3’; *MMP-2*, forward 5’-ATG CTT CCA AAC TTC ACG CTC T-3’ and reverse 5’-CAC AGC CAA CTA CGA TGA CGA-3’; *MT1-MMP*, forward 5’-GGA TAC CCA ATG CCC ATT GGC CA-3’ and reverse 5’-CCA TTG GGC ATC CAG AAG AGA GC-3’; *TIMP-1*, forward 5’-TGC AGG ATG GAC TCT TGC AC-3’ and reverse 5’-TCC AGG GAG CCA CAA AAC TG-3’; *TIMP-2*, forward 5’-GAT GCA CAT CAC CCT CTG TG-3’ and reverse 5’-GTG CCC GTT GAT GTT CTT CT-3’; *Bax*, forward 5’-TTT GCT TCA GGG TTT CAT CC-3’ and reverse 5’-GCC ACT CGG AAA AAG ACC TC-3’; *Bcl-xL*, forward 5’-CAG AGC TTT GAA CAG GTA G-3’ and reverse 5’-GCT CTC GGG TGC TGT ATT G-3’. Glyceraldehyde-3-phosphate dehydrogenase (*GAPDH)* as a control gene was amplified using forward 5’-GTG AAC CAT GAG AAG TAT GAC AA-3’ and reverse 5’-CAT GAG TCC TTC CAC GAT AC-3’ primers. The reactions were carried out using 40 cycles of 95 °C for 10 s (denaturation), 60–62 °C (depending on amplified gene) for 15 s (annealing) and 72 °C for 20 s (elongation). After the real-time PCR, the melting curve was rune to check the specificity of each amplification. The relative expression of messenger RNA (mRNA) in each sample was calculated with the use of the delta–delta cycle threshold (Ct) method [36]. The data were presented as the fold change in target gene expression and normalized to the control gene (*GAPDH*).

### 2.9. Statistical Analysis

In all the experiments, the mean values for three independent assays ± standard deviation (SD) were calculated. The results were subjected to statistical analysis using the one-way analysis of variance (ANOVA) followed by Duncan’s multiple range post hoc test. Differences were recognized as statistically significant at *p* < 0.05. When the one-way analysis of variance revealed any influence of the simultaneous treatment of the human skin fibroblasts with parabens (MP and PP) and RA on the investigated parameter, a two-way analysis of variance (ANOVA/MANOVA, test *F*) was conducted in aim to discern possible independent and/or interactive impact of these agents on this parameter. *F* values having *p* < 0.05 were recognized as statistically significant. Moreover, in the case when the ANOVA/MANOVA analysis disclosed an interactive effect of parabens and RA, the possible character of the interaction was described based on the comparison of the effect of simultaneous treatment with parabens and RA to the sum of effects noted as a result of their separate treatment. The effect of parabens or/and RA was expressed as a factor of change of a measured parameter compared to the control. Based on the performed calculations, it was estimated whether the interaction had an antagonistic (parabens + RA effect < parabens effect + RA effect) or additive (parabens + RA effect = parabens effect + RA effect) character or if it resulted in an enhancement of action (parabens + RA effect > parabens effect + RA effect; synergism or potentiation) [37]. All statistical calculations were performed with the use of statistical software STATISTICA 12 (StatSoft, Tulsa, OK, USA).

## 3. Results

### 3.1. The Influence of RA on Biosynthesis of Collagen in the Fibroblasts Treated with Parabens 

**Figure 2:**
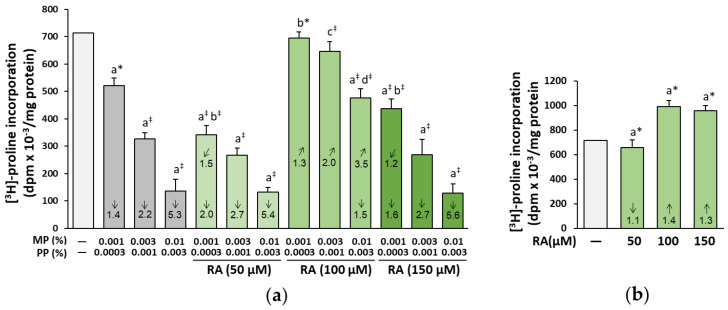
The influence of rosmarinic acid (RA) on the biosynthesis of collagen in the fibroblasts treated with methylparaben (MP) and n-propylparaben (PP) (**a**) and untreated cells (**b**). The mean values ± SD of three experiments done in duplicate are presented. Statistically significant differences are marked as: ^a^ vs. control, ^b^ vs. 0.001% MP and 0.0003% PP, ^c^ vs. 0.003% MP and 0.001% PP, ^d^ vs. 0.01% MP and 0.003% PP; *p < 0.05; ^‡^*p* < 0.001. The values in the bars show a factor of change in comparison to the control (↑, increase; ↓, decrease) or to the respective samples treated with parabens alone (MP and PP) (↗, increase; ↙, decrease).

MP in combination with PP at all concentrations used led to a decrease in the biosynthesis of collagen in a dose-dependent manner (Figure 2a). RA at the concentration of 100 µM completely prevented this decrease in the cells treated with parabens at lower concentrations (0.001% MP and 0.0003% PP, and 0.003% MP and 0.001% PP), and partially in the cells treated with 0.01% MP and 0.003% PP. At the concentrations of 50 and 150 µM of RA, no protection or even increased inhibition of collagen biosynthesis compared to both the control and the respective paraben alone treated cells was noted (Figure 2a). The two-way analysis of variance disclosed that the beneficial impact of RA (100 µM) on the biosynthesis of collagen was mainly an effect of its independent action (*F* = 27.47–226.5, *p* < 0.001); however, at the lowest concentrations of parabens (0.001% MP and 0.0003% PP), the protection provided by this polyphenolic compound also resulted from its interaction with parabens (*F* = 10.29, *p* < 0.05; additive action) (Table 1). The negative influence of RA at the concentration of 50 µM on this process was the result of its independent (*F* = 23.85, *p* < 0.01) and interactive action (*F* = 10.45, *p* < 0.05) with parabens (0.001% MP and 0.0003% PP), whereas in the case of the highest concentration of this compound (150 µM), this effect was caused by its interaction (*F* = 43.67, *p* < 0.001) with parabens (0.001% MP and 0.0003% PP) (Table 1).

RA itself at the concentrations of 100 and 150 µM exerted a stimulatory effect on the biosynthesis of collagen, but at the concentration of 50 µM, it had a slight inhibitory effect (Figure 2b).

### 3.2. The Influence of RA on the Expression of Collagen I and III in the Fibroblasts Treated with Parabens 

**Figure 3:**
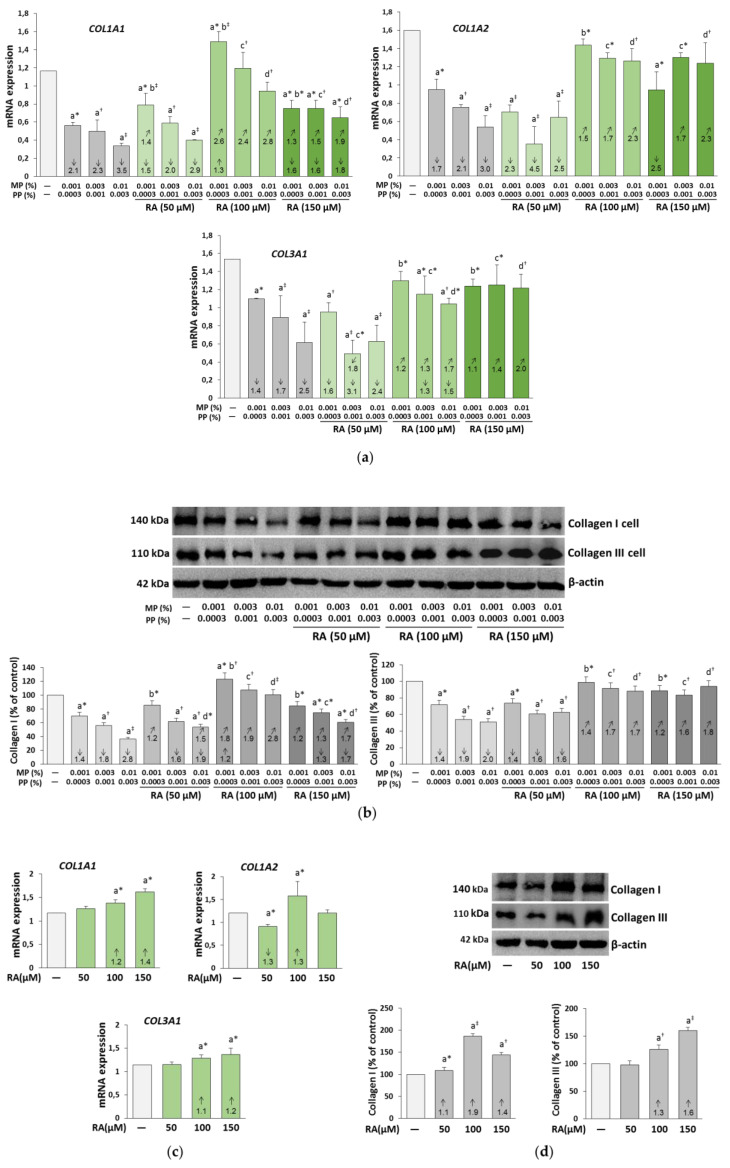
The influence of rosmarinic acid (RA) on the expression of *COL1A1* and *COL1A2* genes coding collagen I, and *COL3A1* coding collagen III (**a**,**c**); on collagen I and III protein (**b**,**d**), in the fibroblasts treated with methylparaben (MP) and n-propylparaben (PP) (**a**,**b**); and untreated cells (**c**,**d**). Expression of genes was assayed by real-time PCR, values represent the mean ± SD of three experiments done in duplicate (**a**,**c**). Representative gels of Western blotting, the intensity of collagen bands was quantified by densitometry and normalized to β-actin, values represent the mean (% of control) ± SD of three experiments (**b**,**d**). Statistically significant differences are marked as: ^a^ vs. control, ^b^ vs. 0.001% MP and 0.0003% PP, ^c^ vs. 0.003% MP and 0.001% PP, ^d^ vs. 0.01% MP and 0.003% PP; *p < 0.05; ^†^*p* < 0.01; ^‡^*p* < 0.001. The values in the bars show a factor of change in comparison to the control (↑, increase; ↓, decrease) or to the respective samples treated with parabens alone (MP and PP) (↗, increase; ↙, decrease).

Expression of *COL1A1* and *COL1A2* genes coding collagen type I and *COL3A1* gene coding collagen type III was inhibited by parabens in a concentration-dependent manner (Figure 3a). In the presence of RA, especially at its higher concentrations (100 and 150 μM), the differences between levels of collagen in paraben-treated and control fibroblasts were diminished. RA at both concentrations caused a significant increase in the expression of collagen genes in relation to the respective samples treated with parabens alone. RA at the concentration of 100 μM normalized expression of both genes of collagen type I in the cells exposed to parabens at all concentrations, and even increased *COL1A1* expression by about 30% as compared to the control in the cells treated with the lowest concentration of parabens (0.001% MP and 0.0003% PP). In contrast to collagen type I, total protection of collagen type III mRNA against negative influence of all concentrations of parabens was revealed at 150 μM RA, while this compound at the concentration of 100 μM completely prevented the inhibitory effect of these preservatives only at their lowest concentrations (0.001% MP and 0.0003% PP). The simultaneous treatment of the fibroblasts with 50 μM RA resulted in a significant increase only in the expression of *COL1A1* gene in the cells exposed to the lowest concentrations of parabens (0.001% MP and 0.0003% PP).

In other cases, 50 μM RA did not affect collagen gene expression in paraben-treated cells except for the decrease in *COL3A1* gene expression in the cells treated with 0.003% MP and 0.001% PP. RA alone stimulated the expression of all collagen genes at the concentration of 100 μM, and *COL1A1* and *COL3A1* at the concentration of 150 μM (Figure 3c). At the lowest concentration (50 μM), RA did not influence the expression of *COL1A1* and *COL3A1* genes, but inhibited that of *COL1A2* gene.

Similarly to the expression of collagen genes, the protein levels of both types of collagen were lower in the paraben-treated fibroblasts in comparison to the non-treated cells, whereas 100 μM RA completely prevented the paraben-induced decrease in the expression of both types of collagen (Figure 3b). Furthermore, an increase in the expression of collagen type I as compared to the control in the cells treated with the lowest concentration of parabens (0.001% MP and 0.0003% PP) was demonstrated. Total or partial protection of collagen type I was also provided by RA at the concentrations of 50 and 150 μM. The decrease in collagen type III expression was rescued with 150 μM RA treatment to 100% of the non-treated cells, which was consistent with the results of *COL3A1* gene expression. In turn, 50 μM RA did not change the expression of collagen III in paraben-treated cells (Figure 3b). The increase in the expression of both collagens was observed under the influence of all concentrations of RA alone, except for no effect of 50 μM RA on collagen III (Figure 3d).

In turn, RA at the concentration of 50 μM did not change the expression of collagen III in paraben-treated cells (Figure 3b). The increase in the expression of both collagen types was observed under the influence of all concentrations of RA alone except for no effect of 50 μM RA on the expression of collagen III (Figure 3d).

The ANOVA/MANOVA analysis disclosed that the beneficial impact of RA on the expression of genes coding collagen type I and III and the protein levels of both types of collagen in the fibroblasts treated with parabens was the result of independent action of this polyphenolic compound (*F* = 5.087–1253, *p* < 0.05–0.001) and its interaction with parabens (*F* = 6.102–1250, *p* < 0.05–0.001), which often was antagonistic in character (Table 2 and Table S2). The 50 μM RA-caused intensification of the decrease in the expression of *COL3A1* gene in the fibroblasts treated with 0.003% MP and 0.001% PP resulted from an independent impact of this compound (*F* = 35.38, *p* < 0.001) (Table S2).

### 3.3. The Influence of RA on the Content of Collagen I and III in the Medium of the Fibroblasts Treated with Parabens

**Figure 4:**
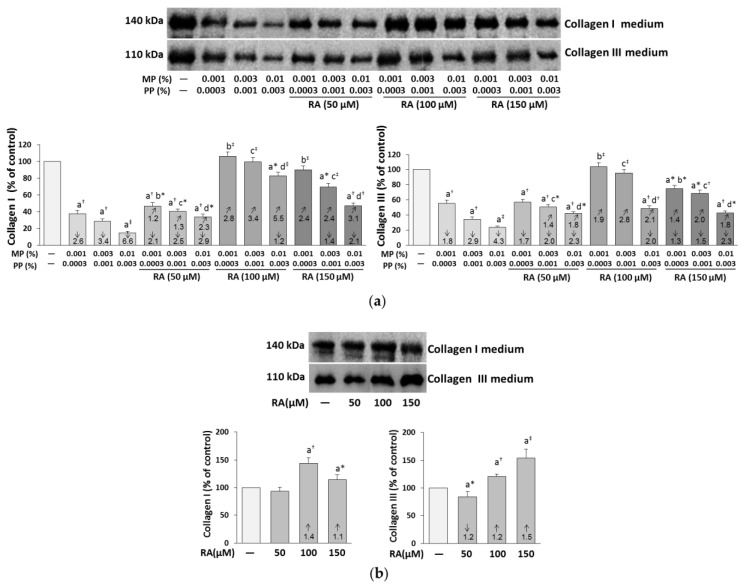
The influence of rosmarinic acid (RA) on the collagen type I and III secreted into media by the fibroblasts treated with methylparaben (MP) and n-propylparaben (PP) (**a**), and untreated cells (**b**). Representative gels of Western blotting, the intensity of the collagen bands was quantified by densitometry, values represent the mean (% of control) ± SD of three experiments. Statistically significant differences are marked as: ^a^ vs. control, ^b^ vs. 0.001% MP and 0.0003% PP, ^c^ vs. 0.003% MP and 0.001% PP, ^d^ vs. 0.01% MP and 0.003% PP; *p < 0.05; ^†^*p* < 0.01; ^‡^*p* < 0.001. The values in the bars show a factor of change in comparison to the control (↑, increase; ↓, decrease) or to the respective samples treated with parabens alone (MP and PP) (↗, increase).

The amount of the extracellular collagen I and III was much lower in the paraben-treated cells than in the control ones, and the addition of RA entirely or partially prevented the deficit of both types of collagen (Figure 4a).

RA at the concentration of 100 μM provided complete protection against the inhibitory effect of the lower concentrations of parabens (0.001% MP and 0.0003% PP, and 0.003% MP and 0.001% PP) on the content of both collagen types. Similar effect on the content of collagen type I in the medium of the cells treated with 0.001% MP and 0.0003% PP was exerted by 150 μM RA.

The above described impact of RA on the content of collagen I and III in the medium of the fibroblasts treated with parabens was caused by independent action of this compound (*F* = 6.228–160.0, *p* < 0.05–0.001) and its interaction with parabens (*F* = 5.207–27.17, *p* < 0.05–0.001). In most cases, this interaction seemed to be antagonistic in character; however, in some cases it was impossible to describe the possible character of the RA–parabens interaction (Table 2). An increase in the collagen I and III levels was demonstrated in the media of the cells treated with RA alone at the concentrations of 100 and 150 μM, whereas 50 μM RA did not influence extracellular collagen I and diminished collagen III levels (Figure 4b).

### 3.4. The Influence of RA on the Expression of ERK1/2 in the Fibroblasts Treated with Parabens

**Figure 5:**
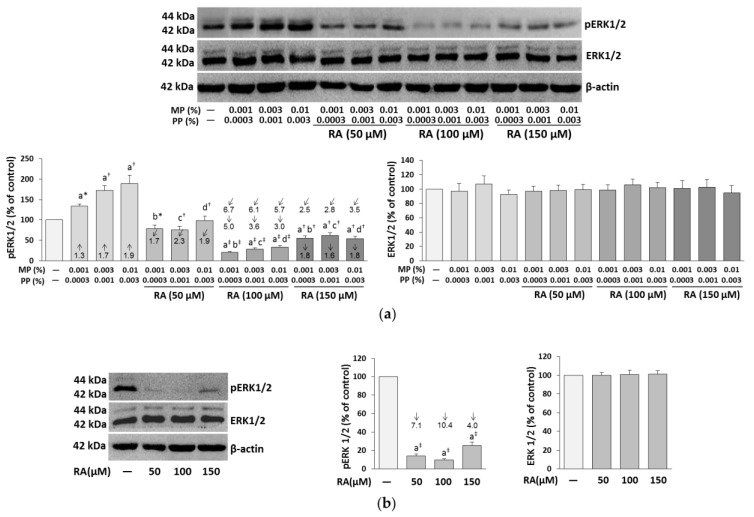
The influence of rosmarinic acid (RA) on the expression of phosphorylated ERK1/2 (pERK1/2) and total ERK1/2 in the fibroblasts treated with methylparaben (MP) and n-propylparaben (PP) (**a**), and untreated cells (**b**). Representative gels of Western blotting, the intensity of the bands was quantified by densitometry and normalized to β-actin, values represent the mean (% of control) ± SD of three experiments. Statistically significant differences are marked as: ^a^ vs. control, ^b^ vs. 0.001% MP and 0.0003% PP, ^c^ vs. 0.003% MP and 0.001% PP, ^d^ vs. 0.01% MP and 0.003% PP; *p < 0.05; ^†^*p* < 0.01; ^‡^*p* < 0.001. The values in the bars show a factor of change in comparison to the control (↑, increase; ↓, decrease) or to the respective samples treated with parabens alone (MP and PP) (↙, decrease).

In the fibroblasts incubated with parabens alone, a significant increase in the level of phosphorylated ERK1/2 (pERK1/2) was found, whereas the addition of RA to these cells at the concentrations of 50, 100, and 150 μM significantly decreased the phosphorylation of these kinases (Figure 5a).

However, RA only at the concentration of 50 μM normalized the phosphorylation of these kinases, but at the higher concentrations (100 and 150 μM), it decreased the kinase phosphorylation not only in relation to the respective samples treated with parabens alone, but also to the control samples.

The ability of RA at the lowest concentration used to have entire protection from paraben-induced inhibition of ERK1/2 phosphorylation was the main effect of the independent action of this polyphenolic compound (*F* = 195.4–391.3, *p* < 0.001). Moreover, the expression of pERK1/2 in the case of the lowest concentrations of parabens (0.001% MP and 0.0003%PP) was also influenced by an antagonistic interaction of RA and parabens (*F* = 19.21, p < 0.01). The inhibitory impact of RA at the concentrations of 100 and 150 μM on the phosphorylation of ERK1/2 in the fibroblasts treated with parabens was the result of both independent (*F* = 128.6–1247, *p* < 0.001) and interactive (*F* = 9.480–264.2, *p* < 0.05–0.001) actions of this compound (Table 1). RA alone also showed strong inhibitory impact on the phosphorylation of ERK1/2 compared to the control (Figure 5b). In turn, the expression of proteins of ERK1/2 did not change in the fibroblasts incubated with the tested compounds as compared to the untreated cells (Figure 5a,b). 

### 3.5. The Influence of RA on the Expression of CTHRC1 and HSP47 in the Fibroblasts Treated with Parabens

**Figure 6:**
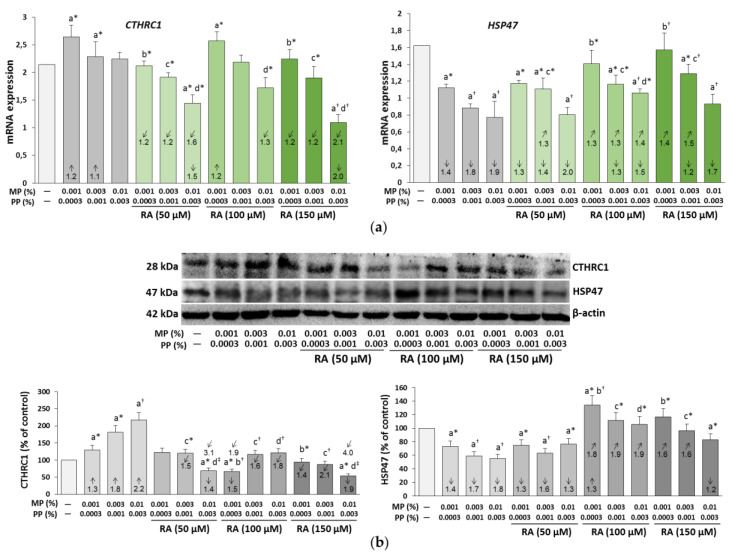

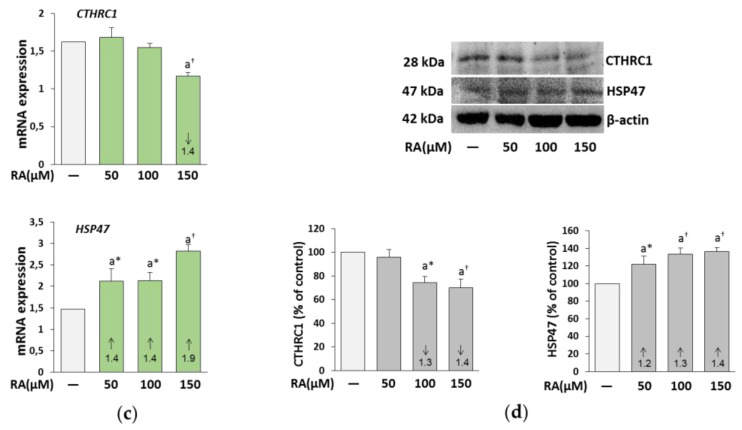
The influence of rosmarinic acid (RA) on the expression of Collagen Triple Helix Repeat Containing-1 (CTHRC1) and Heat shock protein, 47 kDa (HSP47) at mRNA (**a**,**c**); and the protein (**b**,**d**) levels in the fibroblasts treated with methylparaben (MP) and n-propylparaben (PP) (**a**,**b**), and untreated cells (**c**,**d**). Expression of genes was assayed by real-time PCR, values represent the mean ± SD of three experiments done in duplicate (**a**,**c**). Representative gels of Western blotting, the intensity of the bands was quantified by densitometry and normalized to β-actin, values represent the mean (% of control) ± SD of three experiments (**b**,**d**). Statistically significant differences are marked as: ^a^ vs. control, ^b^ vs. 0.001% MP and 0.0003% PP, ^c^ vs. 0.003% MP and 0.001% PP, ^d^ vs. 0.01% MP and 0.003% PP; *p < 0.05; ^†^*p* < 0.01; ^‡^*p* < 0.001. The values in the bars show a factor of change in comparison to the control (↑, increase; ↓, decrease) or to the respective samples treated with parabens alone (MP and PP) (↗, increase; ↙, decrease).

The increase in the expression of CTHRC1 was showed in the paraben-treated fibroblasts at both mRNA (apart from the cells treated with 0.01% MP and 0.003% PP) (Figure 6a) and protein (Figure 6b) levels. RA, depending on its concentration and the concentrations of parabens, exerted either a protective or inhibitory effect as compared to both the control and the respective paraben-treated cells. In the presence of RA alone, no effect or a decrease in the expression of this protein at 100 μM (CTHRC1 protein) and 150 μM (CTHRC1 mRNA and protein) was noted (Figure 6c,d). 

The treatment of fibroblasts with parabens significantly weakened the expression of Hsp47 at both mRNA (Figure 6a) and protein (Figure 6b) levels. RA at the concentration of 50 μM partially protected only the inhibitory effect of 0.003% MP and 0.01% PP on HSP47 mRNA, while at the higher concentrations (100 and 150 μM) it diminished, to a different degree, the inhibitory effect of all concentrations of parabens (except for 0.01% MP and 0.003% PP) on HSP47 mRNA and HSP47 protein. RA alone showed ability to enhance the expression of this protein at both levels at all concentrations (50, 100, and 150 μM) (Figure 6c,d). The ANOVA/MANOVA analysis revealed that the above described impact of RA on the expression of CTHRC1 and HSP47 in the fibroblasts treated with parabens was the result of independent influence of this polyphenolic compound (*F* = 8.316–1321; *p* < 0.05–0.001 and *F* = 6.570–602.1; *p* < 0.05–0.001, respectively) and/or its interactive action with parabens (*F* = 6.447–1297; *p* < 0.05–0.001 and *F* = 7.167–592.6; *p* < 0.001, respectively). It seems that the interaction of RA–parabens in most cases was antagonistic in character, but also additive interactions were disclosed and in some cases other, but impossible to be estimated in character, interactions might occur (Table S3).

### 3.6. The Influence of RA on the Expression and Activity of MMP-1, MMP-2, and MT1-MMP in the Fibroblasts Treated with Parabens 

**Figure 7:**
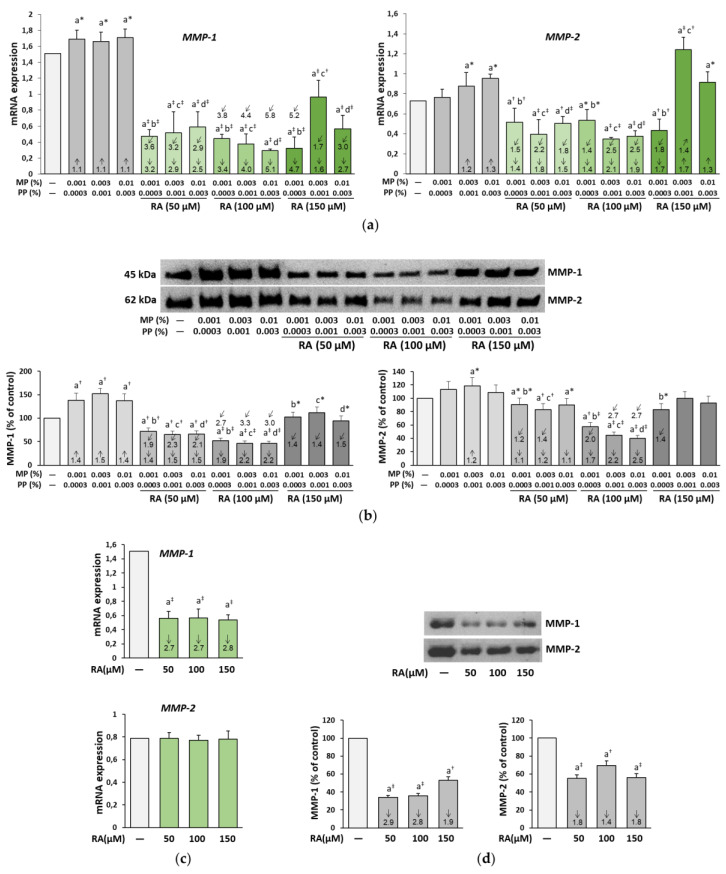
The influence of rosmarinic acid (RA) on the expression of matrix metalloproteinases (MMP-1 and MMP-2) at the level of mRNA (**a**,**c**) and protein (**b**,**d**) in the fibroblasts treated with methylparaben (MP) and n-propylparaben (PP) (**a**,**b**), and untreated cells (**c**,**d**). Expression of genes was assayed by real-time PCR, values represent the mean ± SD of three experiments done in duplicate (**a**,**c**). Representative gels of Western blotting (**b**,**d**), the intensity of the enzyme bands was quantified by densitometry, values represent the mean (% of control) ± SD of three experiments. Statistically significant differences are marked as: ^a^ vs. control, ^b^ vs. 0.001% MP and 0.0003% PP, ^c^ vs. 0.003% MP and 0.001% PP, ^d^ vs. 0.01% MP and 0.003% PP; *p < 0.05; ^†^*p* < 0.01; ^‡^*p* < 0.001. The values in the bars show a factor of change in comparison to the control (↑, increase; ↓, decrease) or to the respective samples treated with parabens alone (MP and PP) (↗, increase; ↙, decrease).

The expression of MMP-1 mRNA and MMP-1 protein was up-regulated under the influence of all concentrations of parabens in contrast to MMP-2, in which mRNA expression was increased in the fibroblasts incubated with the higher concentrations of parabens (0.003% MP and 0.001% PP, and 0.01% MP and 0.003% PP), and the protein expression was increased only in the cells treated with the middle concentration of these compounds (0.003% MP and 0.001% PP) (Figure 7a). The simultaneous treatment of the cells with RA at the concentrations of 50 and 100 μM not only prevented these increases in mRNA levels of both enzymes but caused their significant inhibition in relation to the control. At the highest concentration of RA (150 μM), similar down-regulation of MMP-1 mRNA was found in the cells treated with all concentrations of parabens and of MMP-2 in cells treated with the lowest concentrations of MP and PP. No protection against the stimulating effect of the higher concentrations of parabens (0.003% MP and 0.001% PP, and 0.01% MP and 0.003% PP) on MMP-2 mRNA was noted, but even intensification of the expression by 0.003% MP and 0.001% PP occurred.

In the presence of RA alone, significant inhibition of the expression of MMP-1 mRNA and a lack of an effect on that of MMP-2 mRNA were shown (Figure 7c). The expression of MMP-1 protein was also increased under the influence of all concentrations of parabens, but in contrast to the expression of the enzyme mRNA, RA at the concentration of 150 μM entirely prevented this increase (Figure 7b). RA at the lower concentrations (50 and 100 μM) decreased the expression of this protein as compared to the respective paraben-treated cells, but also to the control ones. The increase in the expression of MMP-2 protein was only seen in the cells treated with 0.003% MP and 0.001% PP, but in contrast to MMP-2, only partial protection was provided by RA. RA itself inhibited (to different degrees) the expression of protein of both enzymes (Figure 7d).

The impact of RA on the expression of MMP-1 mRNA in the paraben-treated fibroblasts mainly resulted from its independent action (*F* = 33.91–1001; *p* < 0.001), while the influence on the expression of MMP-2 mRNA was caused by both independent action of this polyphenolic compound (*F* = 7.889–157.5; *p* < 0.05–0.001) and its interaction with parabens (*F* = 7.140–47.45; *p* < 0.05–0.001) (Table S4). The two-way analysis of variance revealed that the complete protection against the paraben-induced increase in the expression of MMP-1 protein offered by RA at the concentration of 150 μM was an effect of its independent action (*F* = 75.68–106.5; *p* < 0.001), while the protection from the elevation in the expression of MMP-2 protein due to the treatment with 0.003% MP and 0.001% PP resulted from both its independent action (*F* = 41.63; *p* < 0.001) and antagonistic interaction with parabens (*F* = 6.415; *p* < 0.05) (Table S4).

**Figure 8:**
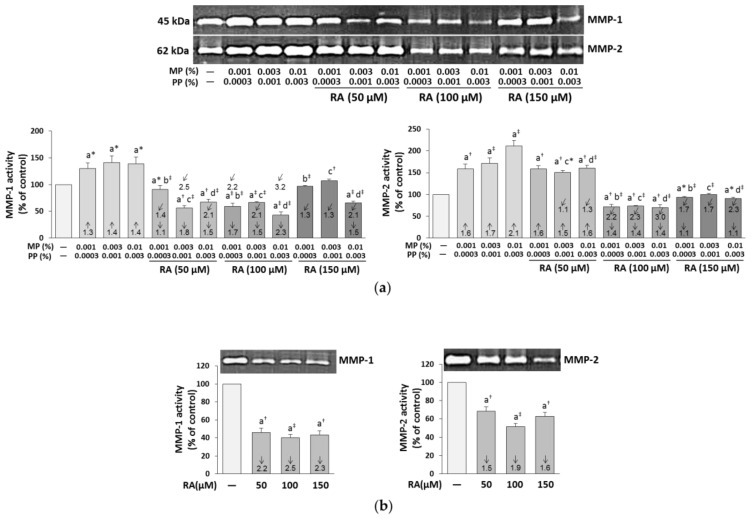
The influence of rosmarinic acid (RA) on the activity of matrix metalloproteinases (MMP-1 and MMP-2) in the fibroblasts treated with methylparaben (MP) and n-propylparaben (PP) (**a**) and untreated cells (**b**). Representative gels of zymography, the intensity of the enzyme bands was quantified by densitometry, values represent the mean (% of control) ± SD of three experiments. Statistically significant differences are marked as: ^a^ vs. control, ^b^ vs. 0.001% MP and 0.0003% PP, ^c^ vs. 0.003% MP and 0.001% PP, ^d^ vs. 0.01% MP and 0.003% PP; *p < 0.05; ^†^*p* < 0.01; ^‡^*p* < 0.001. The values in the bars show a factor of change in comparison to the control (↑, increase; ↓, decrease) or to the respective samples treated with parabens alone (MP and PP) (↙, decrease).

The paraben-induced increase in the activity of MMP-1 and MMP-2 (assayed by the zymography technique) was totally eliminated by the simultaneous treatment of the cells with RA at the concentration of 150 μM and parabens at the concentrations of 0.001% MP and 0.0003% PP, and 0.003% MP and 0.001% PP (MMP-1) and at the same concentration of RA and 0.001% MP and 0.0003% PP (MMP-2) (Figure 8a). RA at the lower concentrations (50 and 100 μM) decreased the activity of MMP; however, mostly below the control levels. The protective impact of RA at the concentration of 150 μM regarding the impact of parabens on the activities of MMP-1 and MMP-2 was the result of independent impact of this compound (*F* = 178.8–420.0; *p* < 0.001) and its antagonistic interaction with parabens (*F* = 11.64–41.60; *p* < 0.01–*p* < 0.001) (Table S5). RA alone exerted an inhibitory effect on the activity of both enzymes (Figure 8b).

**Figure 9:**
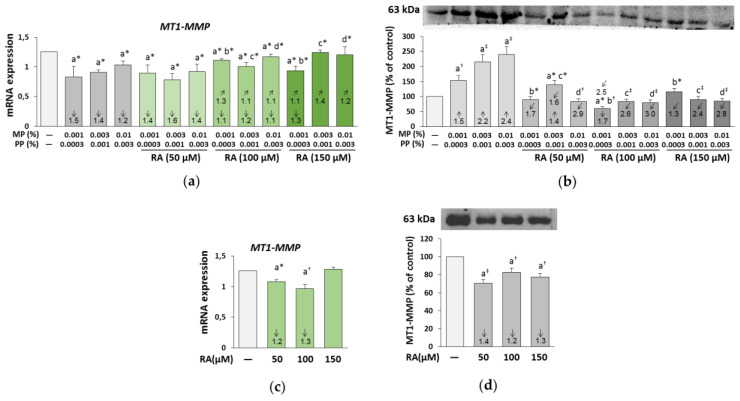
The influence of rosmarinic acid (RA) on the expression of membrane type-1 matrix metalloproteinase (MT1-MMP) at mRNA (**a**,**c**) and the protein (**b**,**d**) levels in the fibroblasts treated with methylparaben (MP) and n-propylparaben (PP) (**a**,**b**), and untreated cells (**c**,**d**). Expression of genes was assayed by real-time PCR, values represent the mean ± SD of three experiments done in duplicate (**a**,**c**). Representative gels of Western blotting, the intensity of the bands was quantified by densitometry and normalized to β-actin, values represent the mean (% of control) ± SD of three experiments (**b**,**d**). Statistically significant differences are marked as: ^a^ vs. control, ^b^ vs. 0.001% MP and 0.0003% PP, ^c^ vs. 0.003% MP and 0.001% PP, ^d^ vs. 0.01% MP and 0.003% PP; *p < 0.05; ^†^*p* < 0.01; ^‡^*p* < 0.001. The values in the bars show a factor of change in comparison to the control (↑, increase; ↓, decrease) or to the respective samples treated with parabens alone (MP and PP) (↗, increase; ↙, decrease).

The expression of MT1-MMP was decreased at the mRNA level (Figure 9a) but increased at the protein level in the fibroblasts treated with parabens (Figure 9b). The beneficial effect of RA at its higher concentrations (100 and 150 μM) on mRNA and at all concentrations on the protein of this enzyme was found in the cells treated with all concentrations of parabens except for the impact of 100 μM RA and 0.001% MP and 0.0003% PP on the MT1-MMP protein. The protective influence of RA against the impact of parabens on the expression of MT1-MMP at the mRNA and protein levels resulted from an independent action of RA (*F* = 24.53–608.2; *p* < 0.01–0.001 and *F* = 26.46–298.4; *p* < 0.001, respectively) and its interaction with parabens (*F* = 6.485–602.6; *p* < 0.05–0.001 and *F* = 5.983–73.85; *p* < 0.05–0.001, respectively) (Table S6). RA alone exerted an inhibitory impact on the enzyme mRNA and protein, except for no effect at 150 μM on MT1-MMP mRNA (Figure 9c,d).

### 3.7. The Influence of RA on the Expression of TIMP-1 and TIMP-2 in the Fibroblasts Treated with Parabens 

**Figure 10:**
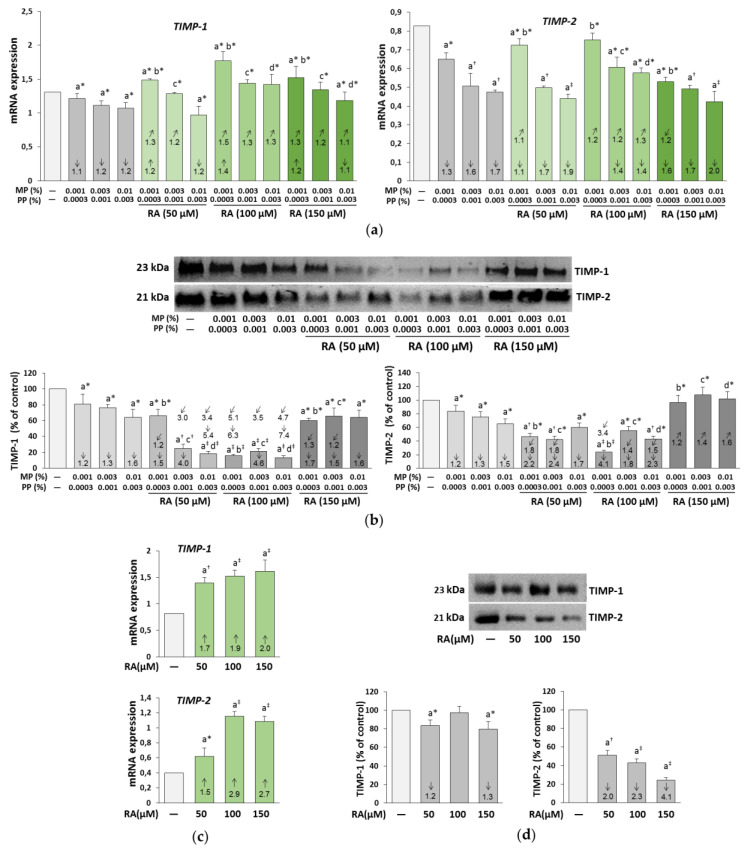
The influence of rosmarinic acid (RA) on the expression of tissue inhibitors of matrix metalloproteinases (TIMP-1 and TIMP-2) at mRNA (**a**,**c**) and protein (**b**,**d**) levels in the fibroblasts treated with methylparaben (MP) and n-propylparaben (PP) (**a**,**b**), and untreated cells (**c**,**d**). Expression of genes was assayed by real-time PCR, values represent the mean ± SD of three experiments done in duplicate (**a**), (**c**). Representative gels of Western blotting (**b**,**d**), the intensity of the bands was quantified by densitometry, values represent the mean (% of control) ± SD of three experiments. Statistically significant differences are marked as: ^a^ vs. control, ^b^ vs. 0.001% MP and 0.0003% PP, ^c^ vs. 0.003% MP and 0.001% PP, ^d^ vs. 0.01% MP and 0.003% PP; *p < 0.05; ^†^*p* < 0.01; ^‡^*p* < 0.001. The values in the bars show a factor of change in comparison to the control (↑, increase; ↓, decrease) or to the respective samples treated with parabens alone (MP and PP) (↗, increase; ↙, decrease).

The expression of TIMP-1 and TIMP-2 was down-regulated by all concentrations of parabens at both mRNA (Figure 10a) and the protein (Figure 10b) levels. The simultaneous treatment of the fibroblasts with RA differentially influenced the expression of the genes and proteins of TIMPs.

The partial or complete protection against alterations in the TIMP genes expression was found at some combinations of concentrations of RA and parabens (Figure 10a) and the impact resulted from an independent action of this polyphenolic compound (*F* = 11.81–267.1; *p* < 0.01–0.001) and its interaction with parabens (*F* = 5.888–262.7; *p* < 0.05–0.001) (Table S6). In contrast, at the protein level, only 150 μM RA was able to entirely prevent changes in the expression of TIMP-2 caused by all concentrations of MP and PP (Figure 10b). This beneficial impact was the result of its independent action (*F* = 27.59–62.23; *p* < 0.001) and antagonistic interaction with parabens (*F* = 127.7–224.8; *p* < 0.001) (Table S7). The acid alone elevated the expression of the genes of both TIMPs (Figure 10c), but reduced the expression of TIMP-1 and TIMP-2 proteins, with one exception of no effect of RA at the concentration of 100 μM on TIMP-1 (Figure 10d).

### 3.8. The Influence of RA on the Proliferation and Viability of the Fibroblasts Treated with Parabens 

**Figure 11:**
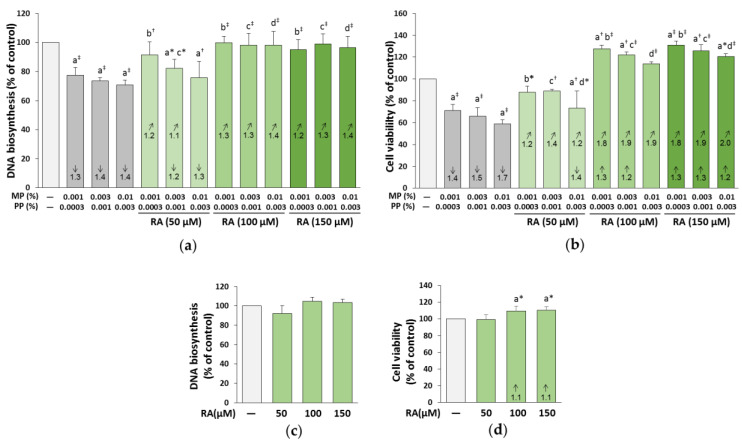
The influence of rosmarinic acid (RA) on the proliferation (**a**,**c**) and the viability (**b**,**d**) of the fibroblasts treated with methylparaben (MP) and n-propylparaben (PP) (**a**,**b**), and untreated cells (**c**,**d**). Values represent the mean ± SD of three experiments done in duplicate. Statistically significant differences are marked as: ^a^ vs. control, ^b^ vs. 0.001% MP and 0.0003% PP, ^c^ vs. 0.003% MP and 0.001% PP, ^d^ vs. 0.01% MP and 0.003% PP; *p < 0.05; ^†^*p* < 0.01; ^‡^*p* < 0.001. The values in the bars show a factor of change in comparison to the control (↑, increase; ↓, decrease) or to the respective samples treated with parabens alone (MP and PP) (↗, increase).

The exposure of the fibroblasts to parabens led to an inhibition of the biosynthesis of DNA by 20%–30% (Figure 11a) and the viability of cells by 30%–40% compared to the control (Figure 11b).

RA at the concentrations of 100 and 150 μM provided total protection of the cells against the inhibition of cell proliferation caused by parabens at all concentrations. RA at the concentration of 50 μM entirely prevented the unfavorable impact of parabens on DNA biosynthesis, but only at the lowest of the concentrations used (0.001% MP and 0.0003% PP). In the case of 0.003% MP and 0.001% PP, the protection offered by this polyphenolic compound was only partial, but at the highest parabens concentration (0.01% MP and 0.003% PP), it did not occur (Figure 11a). The protective impact of RA was the result of its independent action (*F* = 98.63–369.8; *p* < 0.001) and antagonistic interaction with parabens (*F* = 320.6–841.1; *p* < 0.001) (Table 3).

RA at all concentrations also provided protection against the decrease in the viability of fibroblasts caused by parabens at all concentrations; however, dependent on the concentrations of this polyphenolic compound and parabens, this protection was total or partial. RA at the concentration of 150 μM not only entirely prevented the unfavorable impact of parabens (at all concentrations used), but also increased cell survival as compared to the control cells (Figure 11b). The beneficial impact of RA resulted from its independent action (*F* = 8.118–658.7; *p* < 0.05–0.001) and interaction with parabens (*F* = 9.413–324.8; *p* < 0.05–0.001) (Table 3). In some cases, the interaction of RA–parabens seemed to be antagonistic in character, but sometimes it was impossible to evaluate the character of this interaction.

RA alone did not influence the proliferation of fibroblasts at all concentrations used (Figure 11c) and the cell viability at the lowest one (50 μM), but increased the cell survival at the higher concentrations (100 and 150 μM) (Figure 11d).

### 3.9. The Influence of RA on the Expression of Bcl-xL, Bax, and Caspase-3 in the Fibroblasts Treated with Parabens 

**Figure 12:**
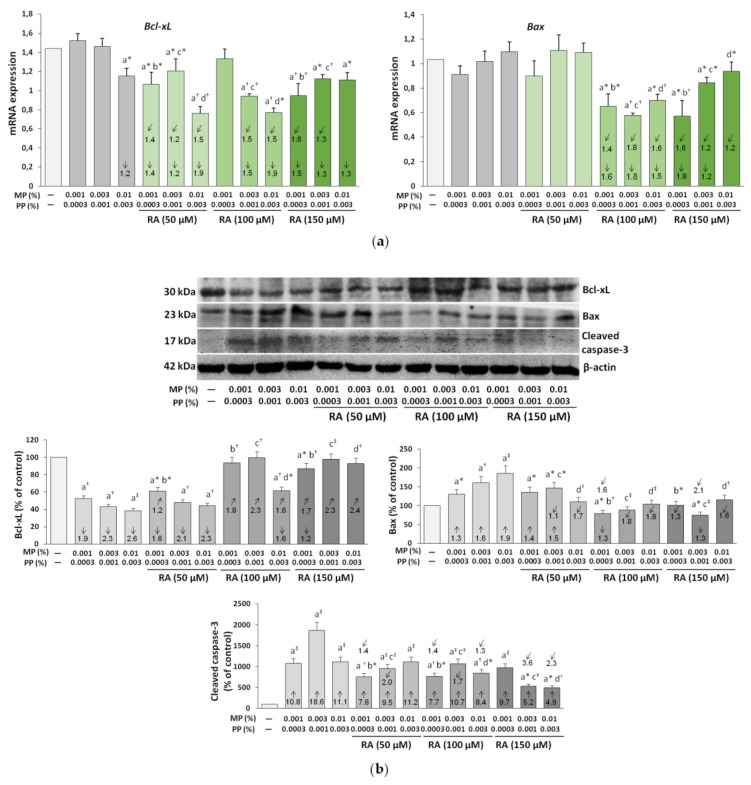

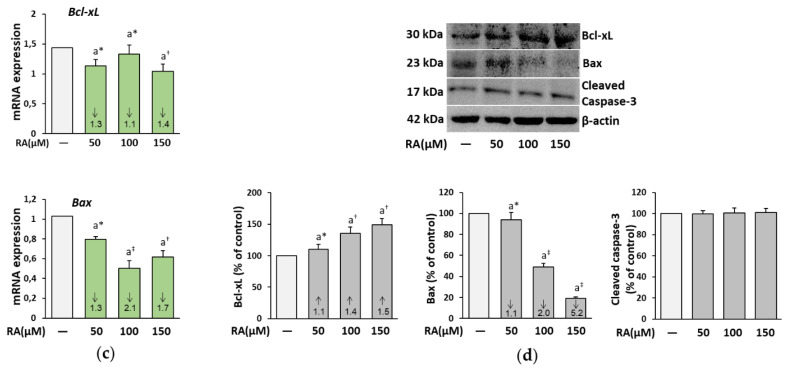
The influence of rosmarinic acid (RA) on the expression of B-cell lymphoma-extra large antiapoptotic protein (Bcl-xL), BCL2-associated X protein (Bax), and caspase-3 at mRNA (**a**,**c**) and protein (**b**,**d**) levels in the fibroblasts treated with methylparaben (MP) and n-propylparaben (PP) (**a**,**b**), and untreated cells (**c**,**d**). Expression of genes was assayed by real-time PCR, values represent the mean ± SD of three experiments done in duplicate (**a**,**c**). Representative gels of Western blotting (**b**,**d**), the intensity of the bands was quantified by densitometry and normalized to β-actin, values represent the mean (% of control) ± SD of three experiments. Statistically significant differences are marked as: ^a^ vs. control, ^b^ vs. 0.001% MP and 0.0003% PP, ^c^ vs. 0.003% MP and 0.001% PP, ^d^ vs. 0.01% MP and 0.003% PP; *p < 0.05; ^†^*p* < 0.01; ^‡^*p* < 0.001. The values in the bars show a factor of change in comparison to the control (↑, increase; ↓, decrease) or to the respective samples treated with parabens alone (MP and PP) (↗, increase; ↙, decrease).

The results presented in Figure 12 showed that parabens differentially affected Bcl-xL and Bax at the mRNA and protein levels. No significant changes, except for a decrease in the expression of Bcl-xL under the influence of 0.01% MP and 0.003% PP, were observed at the mRNA levels (Figure 12a), whereas a significant decrease in the expression of antiapoptotic Bcl-xL, and an increase in the expression of pro-apoptotic Bax were demonstrated at the protein levels (Figure 12b). The presence of the cleaved (active) caspase-3 in the parabens-treated fibroblasts was found (Figure 12b). RA, depending on the concentration, did not influence the expression of Bcl-xL and Bax mRNA and exerted the inhibitory impact as compared to the respective paraben-treated and control cells (Figure 12a). The impact of RA on the expression of Bcl-xL in the fibroblasts treated with parabens mainly was caused by an independent influence of this compound (*F* = 7.084–1086; *p* < 0.05–0.001), whereas the effect on the expression of Bax mRNA resulted from an independent action of RA (*F* = 146.0–600.4; *p* < 0.001) and its interaction with parabens (*F* = 14.08–607.9; *p* < 0.01–0.001) (Table S8). The simultaneous treatment of the cells with RA only partially protected against the paraben-included increase in the expression of the cleaved caspase-3, and depending on the RA and parabens concentrations, partial and total protection of Bcl-xL and Bax proteins was provided by this polyphenolic compound (Figure 12b). The ability of RA to protect the impact of parabens on the expression of the cleaved caspase-3 was determined by an independent action of this compound (*F* = 839.7–6734; *p* < 0.001) and its antagonistic interaction with parabens (*F* = 603.2–16448; *p* < 0.001) (Table S9). The fact that RA was capable of counteracting the impact of parabens on the expression of Bcl-xL mainly resulted from independent influence of this compound (*F* = 11.18–181.1; *p* < 0.05–0.001), whereas the effect on the expression of Bax proteins was caused by independent action of RA (*F* = 82.20–494.5; *p* < 0.001) and in some cases also its antagonistic interaction with parabens (*F* = 12.87–60.04; *p* < 0.01–0.001) (Table S8).

RA alone decreased the expression of Bax at both mRNA (Figure 12c) and protein (Figure 12d) levels, while the expression of Bcl-xL was decreased at the mRNA level (Figure 12c) and increased at the protein level (Figure 12d) under the influence of this compound. No effect of RA alone on the expression of the active caspase-3 was noted (Figure 12d).

## 4. Discussion

This study provides for the first time not only evidence for the unfavorable outcomes of the simultaneous action of two parabens (MP and PP) on the metabolism of collagen in the human skin fibroblasts, but also the protective effect of RA against paraben-induced changes in these cells. Furthermore, this is the first report on the beneficial effect of this polyphenolic compound on the expression and metabolism of this main skin protein in the normal fibroblasts. Moreover, the presented results significantly contributed to clarifying the likely mechanisms of the protective role of RA regarding the toxic action of these two parabens.

MP, in combination with PP, influenced the processes of collagen biosynthesis, secretion, degradation, and regulation, as well as the proliferation and survival of fibroblast. This study has shown that fibroblasts treating with parabens at the concentrations of 0.001% MP and 0.0003% PP, 0.003% MP and 0.001% PP, and 0.01% MP and 0.003% PP can significantly inhibit the biosynthesis of collagen and reduce the proliferation and viability of these cells. Taking into account our previous study on the effect of MP alone on the biosynthesis of collagen in the human skin fibroblasts, it can be concluded that combining these two parabens can intensify the negative effect of MP on this skin protein and on the cellular processes. MP at a concentration 10 times lower than that used in our previous study [28], but in combination with PP (0.001% MP and 0.0003% PP) exerted a similar inhibitory effect (by 32.4%) on this process as 0.01% MP alone (by 27%) [28]. Parabens influenced the biosynthesis of total collagen and the expression of two main types (I and III) of the skin collagen directly at the transcriptional level. In addition, the unfavorable paraben-induced changes in the expression of factors importantly contributing to the regulation of collagen metabolism also suggest an indirect impact of MP and PP on this protein. CTHRC1, which is a 30-kDa protein able to inhibit biosynthesis and deposition of collagen [38], may be involved in the decrease in the collagen biosynthesis and secretion through the paraben-induced up-regulation of this protein. In turn, a down-regulation of the collagen-specific chaperone (HSP47) necessary for stabilization of collagen structure during its secretion [39], can be responsible for the weakened secretion and/or increased degradation of collagen type I and III in paraben-treated cells. Because collagen is not stable even at physiological temperature, HSP47 has two functions: to prevent local unfolding of procollagen and to inhibit the formation of its aggregates. The significance of this collagen chaperone is evidenced by the fact that disruption of the HSP47 gene in mice caused embryonic lethality due to abnormally thin and sensitive to digestion by trypsin collagen fibrils [39]. The significant decrease in the level of the secreted collagen by the paraben-treated fibroblasts may result from increased degradation of collagen because of the diminished level of this chaperone and/or the increased activity of the extracellular MMP-1 and MMP-2. Although MMP-2, known as gelatinase A, is involved in the degradation of collagen type IV, it is also capable to cleave the native fibrillar collagen type I as a specific for this collagen MMP-1 [40]. In addition, MT1-MMP, apart from the role in the regulation of MMP-2 activity and thus indirectly participating in the degradation of collagen, has also the ability to directly degrade type I collagen [41]. Thus, the increase in the expression and/or activity of all of these studied MMPs, associated with the decrease in the expression of their inhibitors (TIMP-1 and TIMP-2), suggest their contribution in increasing collagen degradation and decreasing the level of the extracellular collagen as compared to the control cells. The ERK1/2 signaling pathway is an additional important positive [42] or negative [43], depending on the stimuli and cell types, regulator of collagen expression. The results of our study suggest the negative regulation of collagen biosynthesis with the involvement of ERK1/2 because the significant suppression of production of type I collagen in the paraben-treated fibroblasts negatively correlated with activation (phosphorylation) of these kinases.

These unfavorable effects of parabens on the collagen metabolism in the human skin fibroblasts were associated with impaired proliferation and viability of these cells. The increases in the expression of proapoptotic (Bax, cleaved caspase-3) and decrease in the antiapoptotic (Bcl-xL) markers suggest the possibility of increased apoptosis of the fibroblasts. These results are in accordance with other published reports on the negative effect of MP on the human keratinocytes [30,31,44] and human skin fibroblasts [28,45,46]. In these studies, MP at the concentrations within the range of 0.001%–1.0% exerted the toxic effect on these cells, decreasing their viability, proliferation and increasing the number of apoptotic and necrotic cells [28,30,31,44,45,46]. In addition to the decreased viability and proliferation of the cells, the decrease in the expression of hyaluronan 1 and 2 synthases and type IV collagen at the mRNA level in neonatal human epidermal keratinocytes treated with 0.001% and 0.003% MP was reported [30]. It can therefore be assumed that MP may also have an impact on the synthesis of hyaluronic acid, which together with collagen has the most important functions in the maintaining of skin hydration [15,16,47], but this should be confirmed in future studies. As far as we know, there is one report confirming the negative effect of MP on the skin collagen type I in a mouse model [46]. The topical application of MP (400 mg/mL) to the mouse dorsal skin for 8 weeks (3 times per week) resulted in the skin senescence phenotype, and the skin was dry and pale with an increase in fine wrinkles. The topographic analysis showed an increase in skin roughness in mice treated with MP compared with the control mice. Although the concentration of MP used in this study was overstated than allowed [22,23], the authors believe that MP overdose can cause changes to the ECM components.

MP may also intensify the harmful influence of UVB radiation on the human skin cells, which results in the increase in the production of free radicals, synthesis of nitric oxide, and peroxidation of lipids [31,44]. All these studies, including our results bringing new data on the not known until now probable mechanisms of the action of MP and PP on the skin cells, suggest that long-term skin exposure to parabens alone, as well as to parabens together with UVB, to which a human is exposed during their lifetime, may lead to the development of many different adverse changes in the skin. That is why the searching for protection against all these unfavorable changes caused by parabens is an extremely important part of this research. The discovery of the protective impact of RA regarding the outcomes of paraben action enhances the significance of findings of the study.

Our findings disclosed that this compound is able to prevent or significantly diminish the changes in the metabolism of collagen caused by these preservatives and this protective effect was dependent on the used concentrations of this acid and parabens. Biosynthesis of collagen, as well as the expression of the main skin collagen type I were completely protected from the changes induced by almost all of the used concentrations of parabens by RA at the concentration of 100 μM, while collagen type III was similarly influenced (at the protein level) or even more effectively (at the mRNA level) protected by RA at the concentration of 150 μM. The expression of collagen type III was also higher under the influence of 150 μM than 100 μM RA in the normal cells. In addition to collagen III, RA at the concentration of 150 μM exerted the most beneficial effect on the expression/activity of the enzymes (MMP-1, MMP-2 and MT1-MMP) and MMP-2 inhibitor (TIMP-2) in both paraben-treated and normal cells. It should be underlined, however, that despite the less favorable effect of the acid at the lowest of the concentrations used (50 μM) on collagen biosynthesis and expression, it showed also the protective, to a different degree, effects on some proteins such as CTHRC1, MMP-1, and MMP-2, as well as on the viability and proliferation of paraben-treated cells. A very important finding of the present study is disclosing the enhancement of viability of the normal skin fibroblasts under the influence of RA at the higher concentrations (100 and 150 μM).

Considering a number of other valuable health-promoting properties of RA, the most important in light of our results is its photoprotective activity [9,10,11] due to the fact that it is a strong oxidant [3,4,5,6,7]. RA exerted a significant cytoprotective effect on the keratinocyte cells by reducing UVB-induced intracellular ROS, and weakening oxidative damage to protein and DNA [10]. It has been reported about the benefits of taking orally the preparation containing among others this acid and its positive effect on the health and appearance of the skin [11]. Supplementation for 2 weeks with the extract derived from *Rosmarinus officinalis* L. leaves reduced UV-induced erythema, reduced skin lipoperoxides and wrinkle depth, and increased skin elasticity. The application of cosmetic products containing polyphenolic compounds is also of great importance in reducing transepidermal water loss (TEWL). Emulsions containing RA extracts significantly lowering TEWL improved skin moisture and reduced skin pigmentation [48].

Taking into account detailed findings of the present study and the results of the ANOVA/MANOVA analysis, the beneficial impact of RA on the processes occurring in the fibroblasts, such as the biosynthesis, secretion, and degradation of collagen, as well as proliferation and survival of these cells, may be explained by an independent impact of this polyphenol as well as its interaction with parabens, which often was antagonistic in character. The independent action of this compound on the fibroblasts resulting from its direct influence has been clearly revealed in our study. Because the treatment of fibroblasts with RA has a beneficial impact on the fibroblasts, this compound was also capable of exerting a similar effect in the case of co-treatment with parabens and as a result, the protective effect might occur. However, the protection offered by this polyphenolic compound also resulted from its interaction with parabens. Detailed analysis of the results let us think that the interactive action might be connected with influencing the mechanisms involved in the regulation of the main functions of fibroblasts. It is important to underline that in the case of protective impact of RA, its interaction with parabens seemed to be antagonistic in character, but in some cases it was impossible to recognize the character of the interaction between these agents. Thus, further studies are necessary to understand the possible mechanisms in the interactive action of RA and parabens.

We are aware not only of novelty and achievements of our study, but also of its limitations. The main limitation of our study is the fact that the protective impact of RA towards the unfavorable impact of MP and PP was investigated and revealed at this stage with the use of a simple model of the cultures of fibroblasts of the human skin, and more advanced experimental models such as 3D full-thickness living skin model (3D skin equivalent) constructed with the use of the primary epidermal keratinocytes and dermal fibroblasts would be a better model to extend our future investigations. Moreover, we were unable to recognize with which of the concentrations used, RA provides the most effective protection towards parabens influence on the human skin fibroblasts and how the mechanism of its interactive action with parabens is. However, it needs to be underlined that our study is the first in this subject and its findings provide an important background to plan and perform further comprehensive studies on the possibility of use this polyphenolic compound in protection against their damaging impact on the skin.

## 5. Conclusions

Our results on the simultaneous influence of two widely used parabens such as MP and PP at the concentrations comparable to these used in cosmetics on the human skin fibroblasts confirmed the possibility of an unfavorable impact of these compounds on the skin of cosmetics users and at the same time, enhanced the importance of our study. However, the most important and practically useful finding of our research is revealing that RA is capable of protecting from paraben-induced destroying of such key processes occurring in the fibroblasts as the biosynthesis, secretion, and degradation of collagen, as well as of proliferation and survival of these cells. The beneficial impact of RA on the fibroblasts simultaneously exposed to MP and PP may be explained by the independent action of this compound and its interaction with parabens, which often was antagonistic in character. Based on the results and taking into account the fact that RA is a polyphenolic compound naturally occurring in numerous plants and having confirmed beneficial actions, its seems that this compound may by a good candidate for further studies aimed at finding effective factors allowing a reduction in unfavorable consequences of parabens presence in cosmetics and other preparations applied to the skin.

## Figures and Tables

**Table 1:** Estimation of the main and interactive effects of parabens (methylparaben—MP and propylparaben—PP) and rosmarinic acid (RA) on the biosynthesis of collagen and expression of phosphorylated extracellular signal-regulated kinases (pERK1/2) in the human skin fibroblasts ^1, 2^.

Concentration of RA (μM)	Concentrations of Parabens (%) MP + PP	Parabens (MP + PP) + RA			
		Main Effect of Parabens	Main Effect of RA	Interactive Effect of Parabens—RA	Parabens + RA Effect vs. Parabens Effect + RA Effect *Possible Character of Interaction*
**Biosynthesis of collagen**					
50	0.001 + 0.0003	32.02^‡^	23.85^†^	10.45*	−2 ^3^ vs. −1.4 + (−1.1); −2 vs. −2.5; *Antagonistic action*
	0.003 + 0.001	313.2^‡^	7.276^†^	NS	*No interaction*
	0.01 + 0.003	576.6^‡^	NS	NS	*No interaction*
100	0.001 + 0.0003	33.69^‡^	27.47^‡^	10.29*	0 vs. −1.4 + (+1.4); 0 vs. 0; *Additive action*
	0.003 + 0.001	321.2^‡^	240.1^‡^	NS	*No interaction*
	0.01 + 0.003	706.8^‡^	226.5^‡^	NS	*No interaction*
150	0.001 + 0.0003	84.89^‡^	NS	43.67^‡^	−1.6 vs. −1.4 + (+1.3); −1.6 vs. −0.1 ^4^
	0.003 + 0.001	699.4^‡^	20.49^†^	54.81^‡^	−2.7 vs. −2.2 + (+1.3); −2.7 vs. −0.9 ^4^
	0.01 + 0.003	1278^‡^	35.50^‡^	40.35^‡^	−5.6 vs. −5.3 + (+1.3); −5.6 vs. −4 ^4^
**pERK1/2 expression**					
50	0.001 + 0.0003	192.6^‡^	391.3^‡^	19.21^†^	0 vs. +1.3 + (−7.1); 0 vs. −5.8; *Antagonistic action*
	0.003 + 0.001	168.5^‡^	311.1^‡^	NS	*No interaction*
	0.01 + 0.003	189.2^‡^	195.4^‡^	NS	*No interaction*
100	0.001 + 0.0003	58.05^‡^	1247^‡^	16.43^†^	−5 vs. +1.3 + (−10.4); −5 vs. −9.1; *Antagonistic action*
	0.003 + 0.001	4.208^#^	147.5^‡^	264.2^‡^	−3.6 vs. +1.7 + (−10.4); −3.6 vs. −8.7; *Antagonistic action*
	0.01 + 0.003	NS	128.6^‡^	220.2^‡^	−3 vs. +1.9 + (−10.4); −3 vs. −8.5; *Antagonistic action*
150	0.001 + 0.0003	59.37^‡^	345.0^‡^	NS	*No interaction*
	0.003 + 0.001	91.66^‡^	263.3^‡^	9.480*	−1.6 vs. +1.7 + (−4); −1.6 vs. −2.3; *Antagonistic action*
	0.01 + 0.003	91.91^‡^	288.3^‡^	23.84^†^	−1.8 vs. +1.9 + (−4); −1.8 vs. −2.1; *Antagonistic action*

^1^ The results of the two-way analysis of variance (ANOVA/MANOVA analysis) are presented as *F* values and the level of statistical significance (*p*). *F* values having *p* < 0.05 were recognized as statistically significant (*p < 0.05, ^†^*p* < 0.01, ^‡^*p* < 0.001, ^#^*p* = 0.07). NS—not statistically significant. ^2^ To estimate the possible character of the interaction between parabens (MP and PP) and RA, the effect noted at their simultaneous treatment was compared to the sum of the effects after their separate treatment (parabens and RA effect vs. parabens effect and RA effect). Parabens effect, RA effect, and parabens and RA effect are expressed as factors of changes (−, decrease; +, increase) of a measured parameter in comparison to the control. ^3^ The values represent factors of changes. ^4^ The evaluation of the character of the parabens–RA interaction was impossible.

**Table 2:** Estimation of the main and interactive effects of parabens (methylparaben—MP and propylparaben—PP) and rosmarinic acid (RA) on the expression of intracellular (cell) and extracellular (medium) collagen I and III in the human skin fibroblasts ^1, 2^.

Concentrationof RA (μM)	Concentrations of Parabens (%) MP + PP	Parabens (MP + PP) + RA			
		Main Effect of Parabens	Main Effect ofRA	Interactive Effect of Parabens—RA	Parabens + RA Effect vs. Parabens Effect + RA Effect *Possible Character of Interaction*
**Collagen I cell expression**					
50	0.001 + 0.0003	59.65^‡^	12.47^†^	NS	*No interaction*
	0.003 + 0.001	207.9^‡^	5.415*	NS	*No interaction*
	0.01 + 0.003	393.9^‡^	19.31^†^	NS	*No interaction*
100	0.001 + 0.0003	188.7^‡^	424.8^‡^	24.20^†^	+1.2 ^3^ vs. −1.4 + (+1.9); +1.2 vs. +0.5 ^4^
	0.003 + 0.001	159.3^‡^	90.38^‡^	915.4^‡^	0 vs. −1.8 + (+1.9); 0 vs. +0.1; *Antagonistic action*
	0.01 + 0.003	75.81^‡^	69.71^‡^	1583^‡^	0 vs. −2.8 + (+1.9); 0 vs. −0.9; *Antagonistic action*
150	0.001 + 0.0003	243.4^‡^	99.59^‡^	25.42^‡^	0 vs. −1.4 + (+1.4); 0 vs. 0; *Additive action*
	0.003 + 0.001	560.5^‡^	170.9^‡^	28.82^‡^	−1.3 vs. −1.8 + (+1.4); −1.3 vs. −0.4 ^4^
	0.01 + 0.003	1172^‡^	252.5^‡^	21.27^†^	−1.7 vs. −2.8 + (+1.4); −1.7 vs. −1.4 ^4^
**Collagen I medium expression**					
50	0.001 + 0.0003	102.6^‡^	NS	4.000^#5^	−2.1 vs. −2.6 + 0; −2.1 vs. −2.6; *Antagonistic action*
	0.003 + 0.001	48.80^‡^	NS	NS	*No interaction*
	0.01 + 0.003	303.7^‡^	6.228*	21.01^†^	−2.9 vs. −6.6 + 0; −2.9 vs. −6.6; *Antagonistic action*
100	0.001 + 0.0003	144.8^‡^	89.11^‡^	8.216*	0 vs. −2.6 + (+1.4); 0 vs. −1.2; *Antagonistic action*
	0.003 + 0.001	169.2^‡^	66.34^‡^	NS	*No interaction*
	0.01 + 0.003	274.3^‡^	94.72^‡^	5.207*	−1.2 vs. −6.6 + (+1.4); −1.2 vs. −5.2; *Antagonistic action*
150	0.001 + 0.0003	97.86^‡^	25.87^‡^	NS	*No interaction*
	0.003 + 0.001	200.9^‡^	42.76^‡^	NS	*No interaction*
	0.01 + 0.003	361.1^‡^	52.90^‡^	5.986*	−2.1 vs. −6.6 + (+1.1); −2.1 vs. −5.5; *Antagonistic action*
**Collagen III cell expression**					
100	0.001 + 0.0003	88.74^‡^	80.66^‡^	NS	*No interaction*
	0.003 + 0.001	194.8^‡^	148.6^‡^	35.04^‡^	0 vs. −1.9 + (+1.3); 0 vs. −0.6; *Antagonistic action*
	0.01 + 0.003	646.4^‡^	455.0^‡^	73.99^‡^	0 vs. −2 + (+1.3); 0 vs. −0.7; *Antagonistic action*
150	0.001 + 0.0003	465.6^‡^	281.2^‡^	69.95^‡^	0 vs. −1.4 + (+1.6); 0 vs. 0.2; *Antagonistic action*
	0.003 + 0.001	153.8^‡^	77.97^‡^	16.78^†^	0 vs. −1.9 + (+1.6); 0 vs. −0.3; *Antagonistic action*
	0.01 + 0.003	419.2^‡^	335.3^‡^	9.450*	0 vs. −2 + (+1.6); 0 vs. −0.4; *Antagonistic action*
**Collagen III medium expression**					
50	0.001 + 0.0003	34.77^‡^	10.08*	NS	*No interaction*
	0.003 + 0.001	132.0^‡^	NS	11.33^†^	−2 vs. -2.9 + (−1.2); −2 vs. −4.1; *Antagonistic action*
	0.01 + 0.003	239.5^‡^	NS	10.42*	−2.3 vs. -4.3 + (−1.2); -2.3 vs. −5.5; *Antagonistic action*
100	0.001 + 0.0003	78.34^‡^	28.81*	NS	*No interaction*
	0.003 + 0.001	252.8^‡^	160.0^‡^	NS	*No interaction*
	0.01 + 0.003	415.5^‡^	67.61^‡^	12.57^†^	−2 vs. −4.3 + (+1.2); −2 vs. −3.1; *Antagonistic action*
150	0.001 + 0.0003	100.4^‡^	23.97^†^	27.17^‡^	−1.3 vs. −1.8 + (+1.5); −1.3 vs. −0.3 ^4^
	0.003 + 0.001	180.6^‡^	46.06^‡^	13.22^†^	−1.5 vs. −2.9 + (+1.5); −1.5 vs. −1.4 ^4^
	0.01 + 0.003	289.5^‡^	37.71^‡^	18.37^†^	−2.3 vs. −4.3 + (+1.5); −2.3 vs. −2.8; *Antagonistic action*

^1^ The results of the two-way analysis of variance (ANOVA/MANOVA analysis) are presented as *F* values and the level of statistical significance (*p*). *F* values having *p* < 0.05 were recognized as statistically significant (*p < 0.05, ^†^*p* < 0.01, ^‡^*p* < 0.001). NS—not statistically significant. ^2^ To estimate the possible character of the interaction between parabens (MP and PP) and RA, the effect noted at their simultaneous treatment was compared to the sum of the effects after their separate treatment (parabens and RA effect vs. parabens effect and RA effect). Parabens effect, RA effect, and parabens and RA effect are expressed as factors of changes (−, decrease; +, increase) of a measured parameter in comparison to the control. ^3^ The values represent factors of changes. ^4^ The evaluation of the character of the parabens–RA interaction was impossible. ^5^ Tendency of interactive impact of parabens and RA (^#^*p* = 0.08).

**Table 3:** Estimation of the main and interactive effects of parabens (methylparaben—MP and propylparaben—PP) and rosmarinic acid (RA) on the proliferation, based on [^3^H]thymidine incorporation into deoxyribonucleic acid (DNA), and the viability of the human skin fibroblasts ^1, 2^.

Concentrationof RA (μM)	Concentrations of Parabens (%) MP + PP	Parabens (MP + PP) + RA			
		Main Effect of Parabens	Main Effect ofRA	Interactive Effect of Parabens—RA	Parabens + RA Effect vs. Parabens Effect + RA Effect *Possible Character of Interaction*
**[^3^H]thymidine incorporation**					
50	0.001 + 0.0003	224.1^‡^	369.8^‡^	687.8^‡^	0 vs. −1.3 ^3^ + 0; 0 vs. −1.3; *Antagonistic action*
	0.003 + 0.001	77.71^‡^	244.3^‡^	356.0^‡^	−1.2 vs. −1.4 + 0; −1.2 vs. −1.4; *Antagonistic action*
	0.01 + 0.003	18.44^†^	96.58^‡^	120.5^‡^	−1.3 vs. −1.4 + 0; −1.3 vs. −1.4; *Antagonistic action*
100	0.001 + 0.0003	230.4^‡^	231.2^‡^	643.7^‡^	0 vs. −1.3 + 0; 0 vs. −1.3; *Antagonistic action*
	0.003 + 0.001	229.7^‡^	255.8^‡^	730.6^‡^	0 vs. −1.4 + 0; 0 vs. −1.4; *Antagonistic action*
	0.01 + 0.003	93.16^‡^	104.2^‡^	336.2^‡^	0 vs. −1.4 + 0; 0 vs. −1.4; *Antagonistic action*
150	0.001 + 0.0003	238.3^‡^	322.4^‡^	708.3^‡^	0 vs. −1.3 + 0; 0 vs. −1.3; *Antagonistic action*
	0.003 + 0.001	245.8^‡^	260.7^‡^	841.1^‡^	0 vs. −1.4 + 0; 0 vs. −1.4; *Antagonistic action*
	0.01 + 0.003	78.31^‡^	98.63^‡^	320.6^‡^	0 vs. −1.4 + 0; 0 vs. −1.4; *Antagonistic action*
**Viability of cells**					
50	0.001 + 0.0003	61.54^‡^	13.68^†^	15.83^†^	0 vs. −1.4 + 0; 0 vs. −1.4; *Antagonistic action*
	0.003 + 0.001	95.04^‡^	23.17^†^	26.40^‡^	0 vs. −1.5 + 0; 0 vs. −1.5; *Antagonistic action*
	0.01 + 0.003	93.02^‡^	8.118*	9.413*	−1.4 vs. −1.7 + 0; −1.4 vs. −1.7; *Antagonistic action*
100	0.001 + 0.0003	4.286^#^	207.7^‡^	106.5^‡^	+1.3 vs. −1.4 + (+1.1); +1.3 vs. −0.3 ^4^
	0.003 + 0.001	12.29^†^	241.0^‡^	169.6^‡^	+1.2 vs. −1.5 + (+1.1); +1.2 vs. −0.4 ^4^
	0.01 + 0.003	108.7^‡^	410.6^‡^	288.6^‡^	0 vs. −1.7 + (+1.1); 0 vs. −0.6; *Antagonistic action*
150	0.001 + 0.0003	NS	304.3^‡^	149.6^‡^	+1.3 vs. −1.4 + (+1.1); +1.3 vs. −0.3 ^4^
	0.003 + 0.001	1312^‡^	306.1^‡^	147.3^‡^	+1.3 vs. −1.5 + (+1.1); +1.3 vs. −0.4 ^4^
	0.01 + 0.003	108.7^‡^	658.7^‡^	324.8^‡^	+1.2 vs. −1.7 + (+1.1); +1.2 vs. −0.6 ^4^

^1^ The results of the two-way analysis of variance (ANOVA/MANOVA analysis) are presented as *F* values and the level of statistical significance (p). *F* values having *p* < 0.05 were recognized as statistically significant (*p < 0.05, ^†^*p* < 0.01, ^‡^*p* < 0.001, ^#^*p* = 0.07). NS—not statistically significant. ^2^ To estimate the possible character of the interaction between parabens (MP and PP) and RA, the effect noted at their simultaneous treatment was compared to the sum of the effects after their separate treatment (parabens and RA effect vs. parabens effect and RA effect). Parabens effect, RA effect, and parabens and RA effect are expressed as factors of changes (−, decrease; +, increase) of a measured parameter in comparison to the control. ^3^ The values represent factors of changes. ^4^ The evaluation of the character of the parabens–RA interaction was impossible.

## Supplementary Materials

The following are available online at https://www.mdpi.com/2072-6643/12/5/1282/s1, Table S1: The ratio of the concentrations of parabens (methylparaben—MP and propylparaben—PP) in the selected cosmetic and hygiene products. Table S2: Estimation of the main and interactive effects of parabens (methylparaben—MP and propylparaben—PP) and rosmarinic acid (RA) on the expression of *COL1A1* and *COL1A2* genes coding collagen I, and *COL3A1* coding collagen III in the human skin fibroblasts. Table S3: Estimation of the main and interactive effects of parabens (methylparaben—MP and propylparaben—PP) and rosmarinic acid (RA) on the expression of collagen triple helix repeat containing-1 (CTHRC1) and heat shock protein, 47 kDa (HSP47) at the mRNA and protein levels in the human skin fibroblasts. Table S4: Estimation of the main and interactive effects of parabens (methylparaben—MP and propylparaben—PP) and rosmarinic acid (RA) on the expression of matrix metalloproteinases (MMP-1 and MMP-2) at the mRNA and protein levels in the human skin fibroblasts. Table S5: Estimation of the main and interactive effects of parabens (methylparaben—MP and propylparaben—PP) and rosmarinic acid (RA) on the activity of matrix metalloproteinases (MMP-1 and MMP-2) in the human skin fibroblasts. Table S6: Estimation of the main and interactive effects of parabens (methylparaben—MP and propylparaben—PP) and rosmarinic acid (RA) on the expression of membrane type-1 matrix metalloproteinase (MT1-MMP) at the mRNA and protein levels in the human skin fibroblasts. Table S7: Estimation of the main and interactive effects of parabens (methylparaben—MP and propylparaben—PP) and rosmarinic acid (RA) on the expression of tissue inhibitors of matrix metalloproteinases (TIMP-1 and TIMP-2) at the mRNA and protein levels in the human skin fibroblasts. Table S8: Estimation of the main and interactive effects of parabens (methylparaben—MP and propylparaben—PP) and rosmarinic acid (RA) on the expression of B-cell lymphoma-extra large antiapoptotic protein (Bcl-xL) and BCL2-associated X protein (Bax) at the mRNA and protein levels in the human skin fibroblasts. Table S9: Estimation of the main and interactive effects of parabens (methylparaben—MP and propylparaben—PP) and rosmarinic acid (RA) on the expression of cleaved caspase-3 in the human skin fibroblasts.

## References

[1] Petersen M., Simmonds M.S. (2003). Rosmarinic acid. Phytochemistry.

[2] Shekarchi M., Hajimehdipoor H., Saeidnia S., Gohari A.R., Hamedani M.P. (2012). Comparative study of rosmarinic acid content in some plants of Labiatae family. Pharmacogn. Mag..

[3] Amoah S.K., Sandjo L.P., Kratz J.M., Biavatti M.W. (2016). Rosmarinic acid--pharmaceutical and clinical aspects. Planta Med..

[4] Nieto G., Ros G., Castillo J. (2018). Antioxidant and antimicrobial properties of Rosemary (*Rosmarinus officinalis*, L.): A Review. Medicines.

[5] Osakabe N., Takano H., Sanbongi C., Yasuda A., Yanagisawa R., Inoue K., Yoshikawa T. (2004). Anti-inflammatory and anti-allergic effect of rosmarinic acid (RA); inhibition of seasonal allergic rhinoconjunctivitis (SAR) and its mechanism. Biofactors.

[6] Zou Z.W., Xu L.N., Tian J.Y. (1993). Antithrombotic and antiplatelet effects of rosmarinic acid, a water-soluble component isolated from radix Salviae miltiorrhizae (danshen). Yao Xue Xue Bao.

[7] Radziejewska I., Supruniuk K., Nazaruk J., Karna E., Popławska B., Bielawska A., Galicka A. (2018). Rosmarinic acid influences collagen, MMPs, TIMPs, glycosylation and MUC1 in CRL-1739 gastric cancer cell line. Biomed. Pharmacother..

[8] Choi S.H., Jang G.W., Choi S.I., Jung T.D., Cho B.Y., Sim W.S., Han X., Lee J.S., Kim D.Y., Kim D.B. (2019). Development and validation of an analytical method for carnosol, carnosic acid and rosmarinic acid in food matrices and evaluation of the antioxidant activity of rosemary extract as a food additive. Antioxidants.

[9] Martin R., Pierrard C., Lejeune F., Hilaire P., Breton L., Bernerd F. (2008). Photoprotective effect of a water-soluble extract of Rosmarinus officinalis L. against UV-induced matrix metalloproteinase-1 in human dermal fibroblasts and reconstructed skin. Eur. J. Dermatol..

[10] Fernando P.M.D.J., Piao M.J., Kang K.A., Ryu Y.S., Hewage S.R.K.M., Chae S.W., Hyun J.W. (2016). Rosmarinic acid attenuates cell damage against UVB radiation-induced oxidative stress via enhancing antioxidant effects in human HaCaT cells. Biomol. Ther. (Seoul).

[11] Nobile V., Michelotti A., Cestone E., Caturla N., Castillo J., Benavente-García O., Pérez-Sánchez A., Micol V. (2016). Skin photoprotective and antiageing effects of a combination of rosemary (Rosmarinus officinalis) and grapefruit (Citrus paradisi) polyphenols. Food Nutr. Res..

[12] Richardson M. (2003). Understanding the structure and function of the skin. Nurs. Times.

[13] Gelse K., Poschl E., Aigner T. (2003). Collagens-structure, function, and biosynthesis. Adv. Drug Deliv. Rev..

[14] Avila Rodríguez M.I., Rodríguez Barroso L.G., Sánchez M.L. (2018). Collagen: A review on its sources and potential cosmetic applications. J. Cosmet. Dermatol..

[15] Asserin J., Lati E., Shioya T., Prawitt J. (2015). The effect of oral collagen peptide supplementation on skin moisture and the dermal collagen network: Evidence from an ex vivo model and randomized, placebo-controlled clinical trials. J. Cosmet. Dermatol..

[16] Bolke L., Schlippe G., Gerß J., Voss W.A. (2019). Collagen supplement improves skin hydration, elasticity, roughness, and density: Results of a randomized, placebo-controlled, blind study. Nutrients.

[17] Jadoon S., Karim S., Asad M.H.H.B., Akram M.R., Khan A.K., Malik A., Chen C., Murtaza G. (2015). Anti-aging potential of phytoextract loaded-pharmaceutical creams for human skin cell longetivity. Oxid. Med. Cell. Longev..

[18] Ribeiro A.S., Estanqueiro M., Oliveira M.B., Lobo J.M.S., Benefits M. (2015). Applicability of plant extracts in skin care products. Cosmetics.

[19] Cavinato M., Waltenberger B., Baraldo G., Grade C.V.C., Stuppner H., Jansen-Dürr P. (2017). Plant extracts and natural compounds used against UVB-induced photoaging. Biogerontology.

[20] Soni M.G., Carabin I.G., Burdock G.A. (2005). Safety assessment of esters of p-hydroxybenzoic acid (parabens). Food Chem. Toxicol..

[21] Cowan-Ellsberry C.E., Robison S.H. (2009). Refining aggregate exposure: Example using parabens. Regul. Toxicol. Pharmacol..

[22] EC (2009). Regulation No 1223/2009 of the European Parliament and of the Council of 30 November 2009 on Cosmetic Products.

[23] EC (2014). Regulation No 1004/2014 Amending Annex V to Regulation (EC) No 1223/2009 of the European Parliament and the Council on Cosmetic Products.

[24] Darbre P.D., Harvey P.W. (2008). Paraben esters: Review of recent studies of endocrine toxicity, absorption, esterase and human exposure, and discussion of potential human health risks. J. Appl. Toxicol..

[25] Cashman A.L., Warshaw E.M. (2005). Parabens: A review of epidemiology, structure, alergenicity, and hormonal properties. Dermatitis.

[26] Boberg J., Taxvig C., Christiansen S., Hass U. (2010). Possible endocrine disrupting effects of parabens and their metabolites. Reprod. Toxicol..

[27] Matwiejczuk N., Galicka A., Brzóska M.M. (2020). Review of the safety of application of cosmetic products containing parabens. J. Appl. Toxicol..

[28] Majewska N., Zaręba I., Surażyński A., Galicka A. (2017). Methylparaben-induced decrease in collagen production and viability of cultured human dermal fibroblasts. J. Appl. Toxicol..

[29] Rastogi S.C., Schouten A., de Kruijf N., Weijland J.W. (1995). Contents of methyl-, ethyl-, propyl-, butyl- and benzylparaben in cosmetic products. Contact Dermat..

[30] Ishiwatari S., Suzuki T., Hitomi T., Yoshino T., Matsukuma S., Tsuji T. (2007). Effects of methyl paraben on skin keratinocytes. J. Appl. Toxicol..

[31] Handa O., Kokura S., Adachi S., Takagi T., Naito Y., Tanigawa T., Yoshida N., Yoshikawa T. (2006). Methylparaben potentiates UV-induced damage of skin keratinocytes. Toxicology.

[32] Pedersen S., Marra F., Micoli S., Santi P. (2007). In vitro skin permeation and retention of parabens from cosmetic formulations. Int. J. Cosmet. Sci..

[33] Soni M.G., Taylor S.L., Greenberg N.A., Burdock G.A. (2002). Evaluation of the health aspects of methyl paraben: A review of the published literature. Food Chem. Toxicol..

[34] Peterkofsky B., Chojkier M., Bateman J., Furthmayr H. (1982). Determination of collagen synthesis in tissue and cell culture system. Immuno-Chemistry of the Extracellular Matrix.

[35] Laemmli U.K. (1970). Cleavage of structural proteins during the assembly of the head of bacteriophage T4. Nature.

[36] Livak K.J., Schmittgen T.D. (2001). Analysis of relative gene expression data using real-time quantitative PCR and the 2(-Delta Delta C(T)) Method. Methods.

[37] Mężyńska M., Brzóska M.M., Rogalska J., Galicka A. (2019). Extract from *Aronia melanocarpa* L. berries protects against cadmium-induced lipid peroxidation and oxidative damage to proteins and DNA in the liver: A study in a rat model of environmental human exposure to this xenobiotic. Nutrients.

[38] Zhao M.J., Chen S.Y., Qu X.Y., Abdul-Fattah B., Lai T., Xie M., Wu S.D., Zhou Y.W., Huang C.Z. (2018). Increased Cthrc1 activates normal fibroblasts and suppresses keloid fibroblasts by inhibiting TGF-β/Smad signal pathway and modulating YAP subcellular location. Curr. Med. Sci..

[39] Nagai N., Hosokawa M., Itohara S., Adachi E., Matsushita T., Hosokawa N., Nagata K. (2000). Embryonic lethality of molecular chaperone hsp47 knockout mice is associated with defects in collagen biosynthesis. J. Cell. Biol..

[40] Aimes R.T., Quigley J.P. (1995). Matrix metalloproteinase-2 is an interstitial collagenase. Inhibitor-free enzyme catalyzes the cleavage of collagen fibrils and soluble native type I collagen generating the specific 3/4- and 1/4-length fragments. J. Biol. Chem..

[41] Ohuchi E., Imai K., Fujii Y., Sato H., Seiki M., Okada Y. (1997). Membrane type 1 matrix metalloproteinase digests interstitial collagens and other extracellular matrix macromolecules. J. Biol. Chem..

[42] Park H.J., Ock S.M., Kim H.J., Park H.J., Lee Y.B., Choi J.M., Cho C.S., Lee J.Y., Cho B.K., Cho D.H. (2010). Vitamin C attenuates ERK signalling to inhibit the regulation of collagen production by LL-37 in human dermal fibroblasts. Exp. Dermatol..

[43] Reunanen N., Foschi M., Han J., Kahari V.M. (2000). Activation of extracellular signal-regulated kinase 1/2 inhibits type I collagen expression by human skin fibroblasts. J. Biol. Chem..

[44] Dubey D., Chopra D., Singh J., Srivastav A.K., Kumari S., Verma A., Ray R.S. (2017). Photosensitized methyl paraben induces apoptosis via caspase dependent pathway under ambient UVB exposure in human skin cells. Food Chem. Toxicol..

[45] Carvalho C.M., Menezes P.F., Letenski G.C., Praes C.E., Feferman I.H., Lorencini M. (2012). In vitro induction of apoptosis, necrosis and genotoxicity by cosmetic preservatives: Application of flow cytometry as a complementary analysis by NRU. Int. J. Cosmet. Sci..

[46] Cha H.J., Bae S., Kim K., Kwon S.B., An I.S., Ahn K.J., Ryu J., Kim H., Ye S., Kim B. (2015). Overdosage of methylparaben induces cellular senescence in vitro and in vivo. J. Investig. Dermatol..

[47] Papakonstantinou E., Roth M., Karakiulakis G. (2012). Hyaluronic acid: A key molecule in skin aging. Derm.-Endocrinol..

[48] Cizauskaite U., Bernatoniene J. (2018). Innovative natural ingredients–based multiple emulsions: The effect on human skin moisture, sebum content, pore size and pigmentation. Molecules.

