# Supplementary material for: The Protective Effect of Rosmarinic Acid against Unfavorable Influence of Methylparaben and Propylparaben on Collagen in Human Skin Fibroblasts
Source: Nutrients. 2020 May 1;12(5):1282. doi: 10.3390/nu12051282 (PMC7281983; doi:10.3390/nu12051282)
Supplement: Supplementary file 1 [file nutrients-12-01282-s001.pdf]

nutrients

Suplementary Materials

The Protective Effect of Rosmarinic Acid Against Unfavorable Influence of Methylparaben and Propylparaben on Collagen in Human Skin Fibroblasts

Natalia Matwiejczuk [1,*], Anna Galicka [1,*], Ilona Zaręba [2], Małgorzata M. Brzóska [3]

1 Department of Medical Chemistry, Medical University of Bialystok, Adama Mickiewicza 2A street, 15-222 Bialystok, Poland
2 Department of Medicinal Chemistry, Medical University of Bialystok, Adama Mickiewicza 2D street, 15-222 Bialystok, Poland; ilona.zareba@gmail.com
3 Department of Toxicology, Medical University of Bialystok, Adama Mickiewicza 2C street, 15-222 Bialystok, Poland; malgorzata.brzoska@umb.edu.pl
* Correspondence: natalia.matwiejczuk@umb.edu.pl; angajko@umb.edu.pl; tel.: (4885)7485674, fax: (4885)7485416

Table S1. The ratio of the concentrations of parabens (methylparaben – MP and propylparaben – PP) in the selected cosmetic and hygiene products.

Cosmetic and Hygiene Products	Paraben Concentration	MP and PP Ratio	References
Sun protection cream	MP 308 ± 12 mg/mL<br>PP 112 ± 4 mg/mL	2.75 : 1	[1]
Moisturizing cream	MP 1490 ± 45 mg/mL<br>PP 590 ± 20 mg/mL	2.5 : 1	[1]
Moisturizing cream	MP 1041 ± 35 mg/mL<br>PP 418 ± 20 mg/mL	2.5 : 1	[1]
Moisturizing skin cream	MP 0.42% (w/w)<br>PP 0.14% (w/w)	3 : 1	[2]
Whitening sun protection cream	MP 1370 ± 30 mg/kg<br>PP 435 ± 9 mg/kg	3.1 : 1	[3]
Hand lotion	MP 5.67 ± 0.06% (w/w)<br>PP 1.76 ± 0.08% (w/w	3.2 : 1	[4]
Oil-based lotion	MP 112 μg/mL<br>PP 31 μg/mL	3.6 : 1	[5]
Skin milk	MP 618 mg/kg<br>PP 209 mg/kg	2.95 : 1	[6]
Baby body milk	MP 10.2 μg/g<br>PP 3.21 μg/g	3.2 : 1	[7]
Cleaning mousse (make-up remover)	MP 0.64 ± 0.01 mg/g<br>PP 0.21 ± 0.01 mg/g	3 : 1	[8]
Shampoo	MP 1282 ± 35 mg/mL<br>PP 428 ± 20 mg/mL	3 : 1	[1]
Toothpaste	MP 290 ± 0.5 mg/mL<br>PP 100 ± 0.2 mg/mL	3.6 : 1	[9]

References

1. Márquez-Sillero, I.; Aguilera-Herrador, E.; Cárdenas, S.; Valcárcel, M. Determination of parabens in cosmetic products using multi-walled carbon nanotubes as solid phase extraction sorbent and corona-charged aerosol detection system. *J. Chromatogr. A.* **2010**, *1217*, 1–6. doi: 10.1016/j.chroma.2009.11.005.
2. Msagati, T.A.M.; Barri, T.; Larsson, N.; Jonsson J.A. Analysis and quantification of parabens in cosmetic products by utilizing hollow fibre-supported liquid membrane and high performance liquid chromatography with ultraviolet detection. *Int. J. Cosmet. Sci.* **2008**, *30*, 297–307.
3. Youngvises, N.; Chaida, T.; Khonyoung, S.; Kuppithayanant, N.; Tiyapongpattana, W.; Itharat, A.; Jakmunee, J. Greener liquid chromatography using a guard column with micellar mobile phase for separation of some pharmaceuticals and determination of parabens. *Talanta* **2013**, *106*, 350–359. doi: 10.1016/j.talanta.2012.12.040.
4. Fei, T.; Li, H.; Ding, M.; Ito, M.; Lin, J. Determination of parabens in cosmetic products by solid-phase microextraction of poly(ethylene glycol) diacrylate thin film on fibers and ultra high-speed liquid chromatography with diode array detector. *J. Sep. Sci.* **2011**, *34*, 1599–1606.
5. Han, F.; He, Y.Z.; Yu, C.Z. On-line pretreatment and determination of parabens in cosmetic products by combination of flow injection analysis, solid-phase extraction and micellar electrokinetic Chromatography. *Talanta* **2008**, *74*, 1371–1377. doi: 10.1016/j.talanta.2007.09.007.
6. Lee, M.R.; Lin, C.Y.; Li, Z.G.; Tsai, T.F. Simultaneous analysis of antioxidants and preservatives in cosmetics by supercritical fluid extraction combined with liquid chromatography-mass spectrometry. *J. Chromatogr. A.* **2006**, *1120*, 244–251. doi: 10.1016/j.chroma.2006.01.075.
7. Alvarez-Rivera, G.; Vila, M.; Lores, M.; Garcia-Jares, C.; Llompart, M. Development of a multi-preservative method based on solid-phase micro extraction-gas chromatography-tandem mass spectrometry for cosmetic analysis. *J. Chromatogr. A.* **2014**, *1339*, 13–25. doi: 10.1016/j.chroma.2014.02.075.

8. Ballesta Claver, J.; Valencia, M.C.; Capitán-Vallvey, L.F. Analysis of parabens in cosmetics by low pressure liquid chromatography with monolithic column and chemiluminescent detection. *Talanta* **2009**, *79*, 499–506. doi: 10.1016/j.talanta.2009.04.012.
9. Chuto, M.; Chaiyo, S.; Siangproh, W.; Chailapakul, O. A rapid separation and highly determination of paraben species by ultra-performance liquid chromatography – electrochemical detection. *Detection* **2013**, *1*, 21–29. doi: 10.4236/detection.2013.12004.

Table S2. Estimation of the main and interactive effects of parabens (methylparaben – MP and propylparaben – PP) and rosmarinic acid (RA) on the expression of *COL1A1* and *COL1A2* genes coding collagen I, and *COL3A1* coding collagen III in the human skin fibroblasts. [1, 2.]

Concentration of RA (μM)	Concentrations of Parabens (%) MP + PP	Parabens (MP + PP) + RA			
		Main Effect of Parabens	Main Effect of RA	Interactive Effect of Parabens – RA	Parabens + RA Effect vs. Parabens Effect + RA Effect *Possible Character of Interaction*
			COL1A1 **mRNA expression**		
50	0.001 + 0.0003	60.23‡	24.07†	156.1‡	-1.5 [3] vs. -2.1 + 0; -1.5 vs. -2.1; *Antagonistic action*
	0.003 + 0.001	127.7‡	44.56‡	89.80‡	-2 vs. -2.3 + 0; -2 vs. -2.3; *Antagonistic action*
	0.01 + 0.003	1357‡	238.2‡	399.0‡	-2.9 vs. -3.5 + 0; -2.9 vs. -3.5; *Antagonistic action*
100	0.001 + 0.0003	73.85‡	421.6‡	190.8‡	+1.3 vs. -2.1 + (+1.2); +1.3 vs. -0.9 [4]
	0.003 + 0.001	1253‡	1253‡	1250‡	0 vs. -2.3 + (+1.2); 0 vs. -1.1; *Antagonistic action*
	0.01 + 0.003	1254‡	1253‡	1250‡	0 vs. -3.5 + (+1.2); 0 vs. -2.3; *Antagonistic action*
150	0.001 + 0.0003	915.7‡	177.4‡	33.33‡	-1.6 vs. -2.1 + (+1.4); -1.6 vs. -0.7 [4]
	0.003 + 0.001	508.3‡	109.9‡	10.12*	-1.6 vs. -2.3 + (+1.4); -1.6 vs. -0.9 [4]
	0.01 + 0.003	988.8‡	183.2‡	7.379*	-1.8 vs. -3.5 + (+1.4); -1.8 vs. -2.1; *Antagonistic action*
			COL1A2 **mRNA expression**		
50	0.001 + 0.0003	25.52‡	33.68‡	7.451*	-2.3 vs. -1.7 + (-1.3); -2.3 vs. -3.0; *Antagonistic action*
	0.003 + 0.001	68.36‡	41.04‡	NS	*No interaction*
	0.01 + 0.003	58.05‡	11.01*	20.73†	-2.5 vs. -3 + (-1.3); -2.5 vs. -4.3; *Antagonistic action*
100	0.001 + 0.0003	14.69†	5.087#	6.102*	0 vs. -1.7 + (+1.3); 0 vs. -0.4; *Antagonistic action*
	0.003 + 0.001	34.17‡	NS	25.95‡	0 vs. -2.1 + (+1.3); 0 vs. -0.8; *Antagonistic action*
	0.01 + 0.003	45.86‡	7.847*	33.08‡	0 vs. -3 + (+1.3); 0 vs. -1.7; *Antagonistic action*
150	0.001 + 0.0003	25.77‡	5.008#	4.835# [5]	-2.5 vs. -1.7 + 0; -2.5 vs. -1.7; *Potentiation*
	0.003 + 0.001	23.39†	NS	37.53‡	0 vs. -2.1 + 0; 0 vs. -2.1; *Antagonistic action*
	0.01 + 0.003	31.11‡	NS	35.63‡	0 vs. -3 + 0; 0 vs. -3; *Antagonistic action*
			COL3A1 **mRNA expression**		
50	0.001 + 0.0003	60.81‡	43.78‡	9.329*	-1.6 vs. -1.4 + 0; -1.6 vs. -1.4; *Potentiation*
	0.003 + 0.001	94.26‡	35.38‡	NS	*No interaction*
	0.01 + 0.003	110.6‡	7.754*	8.924*	-2.4 vs. -2.5 + 0; -2.4 vs. -2.5; *Antagonistic action*
100	0.001 + 0.0003	23.57†	NS	36.13‡	0 vs. -1.4 + (+1.1); 0 vs. -0.3; *Antagonistic action*
	0.003 + 0.001	30.18‡	NS	NS	*No interaction*
	0.01 + 0.003	68.32‡	6.805*	4.922#	-1.5 vs. -2.5 + (+1.1); -1.5 vs. -1.4 [4]
150	0.001 + 0.0003	35.60‡	NS	11.25*	0 vs. -1.4 + (+1.2); 0 vs. -0.2; *Antagonistic action*
	0.003 + 0.001	22.72†	NS	10.90*	0 vs. -1.7 + (+1.2); 0 vs. -0.5; *Antagonistic action*
	0.01 + 0.003	56.46‡	9.078*	29.47‡	0 vs. -2.5 + (+1.2); 0 vs. -1.3; *Antagonistic action*

[1] The results of the two-way analysis of variance (ANOVA/MANOVA analysis) are presented as *F* values and the level of statistical significance (*p*). *F* values having $p < 0.05$ were recognized statistically significant (*$p < 0.05$, †$p < 0.01$, ‡$p < 0.001$, #$p = 0.06$). NS – not statistically significant. [2] To estimate the possible character of the interaction between parabens (MP + PP) and RA, the effect noted at their simultaneous treatment was compared to the sum of the effects after their separate treatment (parabens + RA effect vs. parabens effect + RA effect). Parabens effect, RA effect, and parabens + RA effect are expressed as factors of changes (-, decrease; +, increase) of a measured parameter in comparison to the control. [3] The values represent factors of changes. [4] The evaluation of the character of parabens – RA interaction was impossible. [5] Tendency to interactive impact of parabens and RA (#$p = 0.06$).

Table S3. Estimation of the main and interactive effects of parabens (methylparaben – MP and propylparaben – PP) and rosmarinic acid (RA) on the expression of collagen triple helix repeat containing-1 (CTHRC1) and heat shock protein, 47 kDa (HSP47) at the mRNA and protein levels in the human skin fibroblasts. [1, 2.]

Concentration of RA (μM)	Concentrations of Parabens (%) MP + PP	Parabens (MP + PP) + RA			
		**Main Effect of Parabens**	**Main Effect of RA**	**Interactive Effect of Parabens – RA**	**Parabens + RA Effect vs. Parabens Effect + RA Effect** ***Possible Character of Interaction***
			**CTHRC1 mRNA expression**		
50	0.001 + 0.0003	NS	NS	6.975*	0 vs. +1.2 [3] + 0; 0 vs. +1.2; *Antagonistic action*
	0.003 + 0.001	NS	NS	NS	*No interaction*
	0.01 + 0.003	10.58*	NS	NS	*No interaction*
100	0.001 + 0.0003	5.100*	NS	21.15†	+1.2 vs. +1.2 + 0; +1.2 vs. +1.2; *Additive action*
	0.003 + 0.001	1319‡	1321‡	1292‡	0 vs. +1.1 + 0; 0 vs. +1.1; *Antagonistic action*
	0.01 + 0.003	1339‡	1316‡	1297‡	0 vs. 0 + 0; 0 vs. 0 [4]
150	0.001 + 0.0003	5.827*	NS	22.54†	0 vs. +1.2 + (-1.4); 0 vs. -0.2; *Antagonistic action*
	0.003 + 0.001	NS	NS	6.447*	0 vs. +1.1 + (-1.4); 0 vs. -0.3; *Antagonistic action*
	0.01 + 0.003	3.999#	8.316*	NS	*No interaction*
			**CTHRC1 protein expression**		
50	0.001 + 0.0003	19.70†	NS	NS	*No interaction*
	0.003 + 0.001	67.43‡	26.85‡	20.30†	0 vs. +1.8 + 0; 0 vs. +1.8; *Antagonistic action*
	0.01 + 0.003	39.58‡	110.9‡	98.59‡	-1.4 vs. +2.2 + 0; -1.4 vs. +2.2; *Antagonistic action*
100	0.001 + 0.0003	6.581*	106.1‡	18.55†	-1.5 vs. +1.3 + (-1.3); -1.5 vs 0 [4]
	0.003 + 0.001	262.1‡	182.4‡	7.484*	0 vs. +1.8 + (-1.3); 0 vs. +0.5; *Antagonistic action*
	0.01 + 0.003	243.8‡	165.6‡	NS	*No interaction*
150	0.001 + 0.0003	30.05‡	42.31‡	NS	*No interaction*
	0.003 + 0.001	68.94‡	107.6‡	28.46‡	0 vs. +1.8 + (-1.4); 0 vs. +0.4; *Antagonistic action*
	0.01 + 0.003	49.96‡	183.6‡	86.79‡	-1.9 vs. +2.2 + (-1.4); -1.9 vs. +0.8 [4]
			**HSP47 mRNA expression**		
50	0.001 + 0.0003	1089‡	156.8‡	104.9‡	-1.3 vs. -1.4 + (+1.4); -1.3 vs 0 [4]
	0.003 + 0.001	389.6‡	66.96‡	9.548*	-1.4 vs. -1.8 + (+1.4); -1.4 vs. -0.4 [4]
	0.01 + 0.003	879.3‡	54.40‡	40.73‡	-2 vs. -1.9 + (+1.4); -2 vs -0.5 [4]
100	0.001 + 0.0003	215.3‡	89.60‡	7.167*	0 vs. -1.4 + (+1.4); 0 vs. 0; *Additive action*
	0.003 + 0.001	616.8‡	601.5‡	592.4‡	-1.3 vs. -1.8 + (+1.4); -1.3 vs. -0.4 [4]
	0.01 + 0.003	620.8‡	602.1‡	592.6‡	-1.5 vs -1.9 + (+1.4); -1.5 vs. -0.5 [4]
150	0.001 + 0.0003	371.7‡	327.7‡	68.69‡	0 vs. -1.4 + (+1.9); 0 vs. +0.5; *Antagonistic action*
	0.003 + 0.001	539.6‡	269.9‡	66.00‡	-1.2 vs. -1.8 + (+1.9); -1.2 vs. +0.1 [4]
	0.01 + 0.003	1539‡	377.7‡	222.3‡	-1.7 vs. -1.9 + (+1.9); -1.7 vs. 0 [4]
			**HSP47 protein expression**		
50	0.001 + 0.0003	63.35‡	6.570*	NS	*No interaction*
	0.003 + 0.001	115.1‡	8.100*	NS	*No interaction*
	0.01 + 0.003	80.18‡	18.75†	NS	*No interaction*
100	0.001 + 0.0003	10.15*	135.0‡	11.60†	+1.3 vs. -1.4 + (+1.3); +1.3 vs. -0.1 [4]
	0.003 + 0.001	119.9‡	56.26‡	582.4‡	0 vs. -1.7 + (+1.3); 0 vs. -0.4; *Antagonistic action*
	0.01 + 0.003	58.21‡	37.32‡	369.2‡	0 vs. -1.8 + (+1.3); 0 vs. -0.5; *Antagonistic action*
150	0.001 + 0.0003	29.06‡	85.34‡	NS	*No interaction*
	0.003 + 0.001	53.92‡	44.08‡	NS	*No interaction*
	0.01 + 0.003	125.3‡	53.06‡	NS	*No interaction*

[1] The results of the two-way analysis of variance (ANOVA/MANOVA analysis) are presented as F values and the level of statistical significance (p). F values having $p < 0.05$ were recognized statistically significant (*$p < 0.05$, †$p < 0.01$, ‡$p < 0.001$, #$p = 0.08$). NS – not statistically significant. [2] To estimate the possible character of the interaction between parabens (MP + PP) and RA, the effect noted at their simultaneous treatment was compared to the sum of the effects after their separate treatment (parabens + RA effect vs. parabens effect + RA effect). Parabens effect, RA effect, and parabens + RA effect are expressed as factors of changes (-, decrease; +, increase) of a measured parameter in comparison to the control. [3] The values represent factors of changes. [4] The evaluation of the character of parabens – RA interaction was impossible.

Table S4. Estimation of the main and interactive effects of parabens (methylparaben – MP and propylparaben – PP) and rosmarinic acid (RA) on the expression of matrix metalloproteinases (MMP-1 and MMP-2) at the mRNA and protein levels in the human skin fibroblasts. [1, 2.]

**Concentration of RA (μM)**	**Concentrations of parabens (%) MP + PP**	**Parabens (MP + PP) + RA**			
		**Main Effect of Parabens**	**Main Effect of RA**	**Interactive Effect of Parabens – RA**	**Parabens + RA Effect vs. Parabens Effect + RA Effect** ***Possible Character of Interaction***
			**MMP-1 mRNA expression**		
50	0.001 + 0.0003	NS	71.19‡	NS	*No interaction*
	0.003 + 0.001	NS	50.83‡	NS	*No interaction*
	0.01 + 0.003	NS	60.60‡	NS	*No interaction*
100	0.001 + 0.0003	NS	73.32‡	NS	*No interaction*
	0.003 + 0.001	1036‡	1001‡	1041‡	-4 [3] vs. +1.1 + (-2.7); -4 vs. -1.6 [4]
	0.01 + 0.003	1037‡	1000‡	1044‡	-5.1 vs. +1.1 + (-2.7); -5.1 vs. -1.6 [4]
150	0.001 + 0.0003	NS	80.54‡	NS	*No interaction*
	0.003 + 0.001	4.043#	33.91‡	NS	*No interaction*
	0.01 + 0.003	NS	64.43‡	NS	*No interaction*
			**MMP-1 protein expression**		
50	0.001 + 0.0003	84.48‡	258.7‡	NS	-1.4 vs. +1.4 + (-2.9); -1.4 vs. -1.5; *Antagonistic action*
	0.003 + 0.001	180.9‡	602.6‡	11.33†	-1.5 vs +1.5 + (-2.9); -1.5 vs -1.4 [4]
	0.01 + 0.003	85.61‡	331.9‡	NS	*No interaction*
100	0.001 + 0.0003	58.79‡	457.9‡	10.42*	-1.9 vs. +1.4 + (-2.8); -1.9 vs. -1.4 [4]
	0.003 + 0.001	329.8‡	1450‡	NS	*No interaction*
	0.01 + 0.003	147.4‡	780.0‡	NS	*No interaction*
150	0.001 + 0.0003	84.44‡	75.68‡	NS	*No interaction*
	0.003 + 0.001	170.5‡	106.3‡	NS	*No interaction*
	0.01 + 0.003	81.09‡	106.5‡	NS	*No interaction*
			**MMP-2 mRNA expression**		
50	0.001 + 0.0003	11.55†	7.889*	8.126*	-1.4 vs. 0 + 0; -1.4 vs. 0 [4]
	0.003 + 0.001	12.54†	28.70‡	29.10‡	-1.8 vs. +1.2 + 0; -1.8 vs. +1.2 [4]
	0.01 + 0.003	NS	46.68‡	47.45‡	-1.5 vs. +1.3 + 0; -1.5 vs. +1.3 [4]
100	0.001 + 0.0003	10.75*	9.236*	7.140*	-1.4 vs. 0 + 0; -1.4 vs. 0 [4]
	0.003 + 0.001	NS	89.32‡	15.35†	-2.1 vs. +1.2 + 0; -2.1 vs. +1.2 [4]
	0.01 + 0.003	NS	157.5‡	31.90†	-1.9 vs. +1.3 + 0; -1.9 vs. +1.3 [4]
150	0.001 + 0.0003	21.10†	17.17†	15.98†	-1.7 vs. 0 + 0; -1.7 vs. 0 [4]
	0.003 + 0.001	30.64‡	11.97†	12.84†	+1.7 vs. +1.2 + 0; +1.7 vs. +1.2; *Potentiation*
	0.01 + 0.003	11.74†	NS	NS	*No interaction*
			**MMP-2 protein expression**		
50	0.001 + 0.0003	34.22‡	64.78‡	7.062*	-1.1 vs. 0 + (-1.8); -1.1 vs. -1.8; *Antagonistic action*
	0.003 + 0.001	31.32‡	92.58‡	NS	*No interaction*
	0.01 + 0.003	34.97†	53.94‡	9.067*	-1.1 vs. 0 + (-1.8); -1.1 vs. -1.8; *Antagonistic action*
100	0.001 + 0.0003	NS	143.0‡	12.78†	-1.7 vs. 0 + (-1.4); -1.7 vs. -1.4 [4]
	0.003 + 0.001	10.73*	269.0‡	NS	*No interaction*
	0.01 + 0.003	NS	332.4‡	NS	*No interaction*
150	0.001 + 0.0003	22.23†	74.89‡	NS	*No interaction*
	0.003 + 0.001	40.82‡	41.63‡	6.415*	0 vs. +1.2 + (-1.8); 0 vs. -1.8; *Antagonistic action*
	0.01 + 0.003	-	-	-	-

[1] The results of the two-way analysis of variance (ANOVA/MANOVA analysis) are presented as F values and the level of statistical significance (p). F values having $p < 0.05$ were recognized statistically significant (*$p < 0.05$, †$p < 0.01$, ‡$p < 0.001$, #$p = 0.08$). NS – not statistically significant. [2] To estimate the possible character of the interaction between parabens (MP + PP) and RA, the effect noted at their simultaneous treatment was compared to the sum of the effects after their separate treatment (parabens + RA effect vs. parabens effect + RA effect). Parabens effect, RA effect, and parabens + RA effect are expressed as factors of changes (-, decrease; +, increase) of a measured parameter in comparison to the control. [3] The values represent factors of changes. [4] The evaluation of the character of parabens – RA interaction was impossible.

Table S5. Estimation of the main and interactive effects of parabens (methylparaben – MP and propylparaben – PP) and rosmarinic acid (RA) on the activity of matrix metalloproteinases (MMP-1 and MMP-2) in the human skin fibroblasts. [1, 2.]

**Concentration of RA (μM)**	**Concentrations of Parabens (%) MP + PP**	**Parabens (MP + PP) + RA**			
		**Main Effect of Parabens**	**Main Effect of RA**	**Interactive Effect of Parabens – RA**	**Parabens + RA Effect vs. Parabens Effect + RA Effect** ***Possible Character of Interaction***
			**MMP-1 activity**		
50	0.001 + 0.0003	167.9‡	262.1‡	6.288*	-1.1 [3] vs. +1.3 + (-2.2); -1.1 vs. -0.9 [4]
	0.003 + 0.001	108.1‡	803.1‡	39.53‡	-1.8 vs. +1.4 + (-2.2); -1.8 vs. -0.8 [4]
	0.01 + 0.003	335.5‡	1490‡	28.82‡	-1.5 vs. +1.4 + (-2.2); -1.5 vs. -0.8 [4]
100	0.001 + 0.0003	106.5‡	752.2‡	5.333*	-1.7 vs. +1.3 + (-2.5); -1.7 vs. -1.2 [4]
	0.003 + 0.001	51.21‡	400.4‡	71.39‡	-1.5 vs. +1.4 + (-2.5); -1.5 vs. -1.1 [4]
	0.01 + 0.003	NS	445.6‡	120.1‡	-2.3 vs. +1.4 + (-2.5); -2.3 vs. -1.1 [4]
150	0.001 + 0.0003	220.8‡	253.7‡	17.66†	0 vs. +1.3 + (-2.3); 0 vs. -1; *Antagonistic action*
	0.003 + 0.001	238.5‡	178.8‡	11.64†	0 vs. +1.4 + (-2.3); 0 vs. -0.9; *Antagonistic action*
	0.01 + 0.003	275.0‡	1226‡	17.75†	-1.5 vs. +1.4 + (-2.3); -1.5 vs. -0.9 [4]
			**MMP-2 activity**		
50	0.001 + 0.0003	915.5‡	39.94‡	41.42‡	+1.6 vs. +1.6 + (-1.5); +1.6 vs. +0.1 [4]
	0.003 + 0.001	1171‡	135.5‡	5.980*	+1.5 vs. +1.7 + (-1.5); +1.5 vs. +0.2 [4]
	0.01 + 0.003	1211‡	196.7‡	10.57*	+1.6 vs +2.1 + (-1.5); +1.6 vs +0.6 [4]
100	0.001 + 0.0003	289.6‡	854.4‡	69.24‡	-1.4 vs. +1.6 + (-1.9); -1.4 vs. -0.3 [4]
	0.003 + 0.001	395.8‡	901.5‡	51.51‡	-1.4 vs. +1.7 + (-1.9); -1.4 vs. -0.2 [4]
	0.01 + 0.003	540.9‡	1090‡	177.1‡	-1.4 vs. +2.1 + (-1.9); -1.4 vs. +0.2 [4]
150	0.001 + 0.0003	340.0‡	443.3‡	33.30‡	-1.1 vs. +1.6 + (-1.6); -1.1 vs. 0 [4]
	0.003 + 0.001	415.8‡	420.0‡	41.60‡	0 vs. +1.7 + (-1.6); 0 vs. +0.1; *Antagonistic action*
	0.01 + 0.003	582.5‡	741.0‡	206.5‡	-1.1 vs. +2.1 + (-1.6); -1.1 vs. +0.5 [4]

[1] The results of the two-way analysis of variance (ANOVA/MANOVA analysis) are presented as F values and the level of statistical significance (p). F values having $p < 0.05$ were recognized statistically significant (*$p < 0.05$, †$p < 0.01$, ‡$p < 0.001$). NS – not statistically significant. [2] To estimate the possible character of the interaction between parabens (MP + PP) and RA, the effect noted at their simultaneous treatment was compared to the sum of the effects after their separate treatment (parabens + RA effect vs. parabens effect + RA effect). Parabens effect, RA effect, and parabens + RA effect are expressed as factors of changes (-, decrease; +, increase) of a measured parameter in comparison to the control. [3] The values represent factors of changes. [4] The evaluation of the character of parabens – RA interaction was impossible.

Table S6. Estimation of the main and interactive effects of parabens (methylparaben – MP and propylparaben – PP) and rosmarinic acid (RA) on the expression of membrane type-1 matrix metalloproteinase (MT1-MMP) at the mRNA and protein levels in the human skin fibroblasts. [1, 2.]

Concentration of RA (μM)	Concentrations of Parabens (%) MP + PP	Parabens (MP + PP) + RA			
		Main Effect of Parabens	Main Effect of RA	Interactive Effect of Parabens – RA	Parabens + RA Effect vs. Parabens Effect + RA Effect *Possible Character of Interaction*
**MT1-MMP mRNA expression**					
50	0.001 + 0.0003	41.42‡	NS	6.676*	-1.4 [3] vs. -1.5 + (-1.2); -1.4 vs. -2.7; *Antagonistic action*
	0.003 + 0.001	156.9‡	34.55‡	NS	*No interaction*
	0.01 + 0.003	37.23‡	21.81‡	NS	*No interaction*
100	0.001 + 0.0003	16.65‡	NS	49.49‡	-1.1 vs. -1.5 + (-1.3); -1.1 vs. -2.8; *Antagonistic action*
	0.003 + 0.001	619.5‡	607.3‡	602.6‡	-1.2 vs. -1.4 + (-1.3); -1.2 vs. -2.7; *Antagonistic action*
	0.01 + 0.003	612.5‡	608.2‡	601.7‡	-1.1 vs. -1.2 + (-1.3); -1.1 vs. -2.5; *Antagonistic action*
150	0.001 + 0.0003	NS	60.28‡	NS	*No interaction*
	0.003 + 0.001	5.786*	53.69‡	NS	*No interaction*
	0.01 + 0.003	47.73‡	24.53†	6.485*	0 vs. -1.2 + 0; 0 vs. -1.2; *Antagonistic action*
**MT1-MMP protein expression**					
50	0.001 + 0.0003	46.68‡	74.96‡	10.03*	0 vs. +1.5 + (-1.4); 0 vs. +0.1; *Antagonistic action*
	0.003 + 0.001	143.4‡	47.18‡	9.310*	+1.4 vs. +2.2 + (-1.4); +1.4 vs. +0.8 [4]
	0.01 + 0.003	101.3‡	146.0‡	68.00‡	0 vs. +2.4 + (-1.4); 0 vs. +1; *Antagonistic action*
100	0.001 + 0.0003	8.671*	103.4‡	48.56‡	-1.7 vs. +1.5 + (-1.2); -1.7 vs. +0.3 [4]
	0.003 + 0.001	211.5 ‡	287.9‡	5.983*	0 vs. +2.2 + (-1.2); 0 vs. +1; *Antagonistic action*
	0.01 + 0.003	214.6 ‡	298.4‡	16.59†	0 vs. +2.4 + (-1.2); 0 vs. +1.2; *Antagonistic action*
150	0.001 + 0.0003	62.70‡	26.46‡	NS	*No interaction*
	0.003 + 0.001	82.45‡	110.1‡	53.62‡	0 vs. +2.2 + (-1.3); 0 vs. +0.9; *Antagonistic action*
	0.01 + 0.003	92.66‡	132.2‡	73.85‡	0 vs. +2.4 + (-1.3); 0 vs. +1.1; *Antagonistic action*

[1] The results of the two-way analysis of variance (ANOVA/MANOVA analysis) are presented as *F* values and the level of statistical significance (*p*). *F* values having $p < 0.05$ were recognized statistically significant (*$p < 0.05$, †$p < 0.01$, ‡$p < 0.001$). NS – not statistically significant. [2] To estimate the possible character of the interaction between parabens (MP + PP) and RA, the effect noted at their simultaneous treatment was compared to the sum of the effects after their separate treatment (parabens + RA effect vs. parabens effect + RA effect). Parabens effect, RA effect, and parabens + RA effect are expressed as factors of changes (-, decrease; +, increase) of a measured parameter in comparison to the control. [3] The values represent factors of changes. [4] The evaluation of the character of parabens – RA interaction was impossible.

Table S7. Estimation of the main and interactive effects of parabens (methylparaben – MP and propylparaben – PP) and rosmarinic acid (RA) on the expression of tissue inhibitors of matrix metalloproteinases (TIMP-1 and TIMP-2) at the mRNA and protein levels in the human skin fibroblasts. [1,2].

Concentration of RA (μM)	Concentrations of Parabens (%) MP + PP	Parabens (MP + PP) + RA			
		Main Effect of Parabens	Main Effect of RA	Interactive Effect of Parabens – RA	Parabens + RA Effect vs. Parabens Effect + RA Effect *Possible Character of Interaction*
			**TIMP-1 mRNA expression**		
50	0.001 + 0.0003	NS	11.81†	NS	*No interaction*
	0.003 + 0.001	9.116*	11.81†	NS	*No interaction*
	0.01 + 0.003	48.50‡	NS	6.494*	-1.2 [3] vs. -1.2 + (+1.7); -1.2 vs. +0.5 [4]
100	0.001 + 0.0003	NS	54.00‡	7.469*	+1.4 vs. -1.1 + (+1.9); +1.4 vs. +0.8 [4]
	0.003 + 0.001	264.8‡	266.9‡	262.7‡	0 vs. -1.2 + (+1.9); 0 vs. +0.7; *Antagonistic action*
	0.01 + 0.003	265.1‡	267.1‡	262.5‡	0 vs. -1.2 + (+1.9); 0 vs. +0.7; *Antagonistic action*
150	0.001 + 0.0003	NS	60.28‡	NS	*No interaction*
	0.003 + 0.001	82.45‡	110.1‡	53.62‡	0 vs. -1.2 + (+2); 0 vs. +0.8; *Antagonistic action*
	0.01 + 0.003	92.66‡	132.2‡	73.85‡	-1.1 vs. -1.2 + (+2); -1.1 vs. +0.8 [4]
			**TIMP-1 protein expression**		
50	0.001 + 0.0003	16.50†	12.36†	NS	*No interaction*
	0.003 + 0.001	283.6‡	190.5‡	49.70 ‡	-4 vs. -1.3 + (-1.2); -4 vs. -2.5; *Synergistic action*
	0.01 + 0.003	301.9‡	113.9‡	24.83†	-5.4 vs. -1.6 + (-1.2); -5.4 vs. -2.8; *Synergistic action*
100	0.001 + 0.0003	160.0‡	72.10‡	62.20‡	-6.3 vs. -1.2 + 0; -6.3 vs. -1.2; *Potentiation*
	0.003 + 0.001	NS	1567‡	131.7‡	-4.6 vs. -1.3 + 0; -4.6 vs. -1.3; *Potentiation*
	0.01 + 0.003	25.80‡	1039‡	108.9‡	-7.4 vs. -1.6 + 0; -7.4 vs. -1.6; *Potentiation*
150	0.001 + 0.0003	20.27‡	23.46†	NS	*No interaction*
	0.003 + 0.001	60.58‡	42.41‡	NS	*No interaction*
	0.01 + 0.003	40.38‡	6.261*	6.512*	-1.6 vs. -1.6 + (-1.3); -1.6 vs. -2.9; *Antagonistic action*
			**TIMP-2 mRNA expression**		
50	0.001 + 0.0003	NS	NS	5.888*	-1.1 vs. -1.3 + (+1.5); -1.1 vs. +0.2 [4]
	0.003 + 0.001	51.87‡	12.78†	10.96*	-1.7 vs. -1.6 + (+1.5); -1.7 vs. -0.1 [4]
	0.01 + 0.003	70.94‡	15.15†	7.823*	-1.9 vs. -1.7 + (+1.5); -1.9 vs. -0.2 [4]
100	0.001 + 0.0003	69.13‡	38.56‡	10.42*	0 vs. -1.3 + (+2.9); 0 vs. +1.6; *Antagonistic action*
	0.003 + 0.001	333.8‡	139.5‡	77.89‡	-1.4 vs. -1.6 + (+2.9); -1.4 vs. +1.3 [4]
	0.01 + 0.003	427.7‡	162.2‡	90.29‡	-1.4 vs. -1.7 + (+2.9); -1.4 vs. +1.2 [4]
150	0.001 + 0.0003	130.3‡	4.438#	34.70‡	-1.6 vs. -1.3 + (+2.7); -1.6 vs. +1.4; *Antagonistic action*
	0.003 + 0.001	165.0‡	16.80†	11.67†	-1.7 vs. -1.6 + (+2.7); -1.7 vs. +1.1 [4]
	0.01 + 0.003	166.0‡	6.828*	15.23†	-2 vs. -1.7 + (+2.7); -2 vs. +1 [4]
			**TIMP-2 protein expression**		
50	0.001 + 0.0003	11.03*	173.3‡	NS	*No interaction*
	0.003 + 0.001	31.42‡	183.5‡	6.960*	-2.4 vs. -1.3 + (-2); -2.4 vs. -3.3; *Antagonistic action*
	0.01 + 0.003	17.78†	77.49‡	49.73‡	-1.7 vs. -1.5 + (-2); -1.7 vs. -3.5; *Antagonistic action*
100	0.001 + 0.0003	36.75‡	398.1‡	NS	*No interaction*
	0.003 + 0.001	95.28‡	10.34†	6.324*	-1.8 vs. -1.3 + (-2.3); -1.8 vs. -3.6; *Antagonistic action*
	0.01 + 0.003	189.7‡	14.78†	9.641*	-2.3 vs. -1.5 + (-2.3); -2.3 vs. -3.8; *Antagonistic action*
150	0.001 + 0.0003	49.60†	62.23‡	127.7‡	0 vs. -1.2 + (-4.1); 0 vs. -5.3; *Antagonistic action*
	0.003 + 0.001	39.14‡	50.91‡	164.9‡	0 vs. -1.3 + (-4.1); 0 vs. -5.4; *Antagonistic action*
	0.01 + 0.003	32.21‡	27.59‡	224.8‡	0 vs. -1.5 + (-4.1); 0 vs. -5.6; *Antagonistic action*

[1] The results of the two-way analysis of variance (ANOVA/MANOVA analysis) are presented as F values and the level of statistical significance (p). F values having $p < 0.05$ were recognized statistically significant (*$p < 0.05$, †$p < 0.01$, ‡$p < 0.001$, #$p = 0.07$). NS – not statistically significant. [2] To estimate the possible character of the interaction between parabens (MP + PP) and RA, the effect noted at their simultaneous treatment was compared to the sum of the effects after their separate treatment (parabens + RA effect vs. parabens effect + RA effect). Parabens effect, RA effect, and parabens + RA effect are expressed as factors of changes (-, decrease; +, increase) of a measured parameter in comparison to the control. [3] The values represent factors of changes. [4] The evaluation of the character of parabens – RA interaction was impossible.

Table S8. Estimation of the main and interactive effects of parabens (methylparaben – MP and propylparaben – PP) and rosmarinic acid (RA) on the expression of B-cell lymphoma-extra large antiapoptotic protein (Bcl-xL) and BCL2-associated X protein (Bax) at the mRNA and protein levels in the human skin fibroblasts. [1, 2.]

Concentration of RA (μM)	Concentrations of Parabens (%) MP + PP	Parabens (MP + PP) + RA			
		Main Effect of Parabens	Main Effect of RA	Interactive Effect of Parabens – RA	Parabens + RA Effect vs. Parabens Effect + RA Effect *Possible Character of Interaction*
**Bcl-xL mRNA expression**					
50	0.001 + 0.0003	NS	26.14‡	NS	*No interaction*
	0.003 + 0.001	NS	15.80†	NS	*No interaction*
	0.01 + 0.003	15.91†	18.00†	NS	*No interaction*
100	0.001 + 0.0003	-	-	-	-
	0.003 + 0.001	1104‡	1084‡	1105‡	-1.5 [3] vs. 0 + (-1.1); -1.5 vs. -1.1 [4]
	0.01 + 0.003	1113‡	1086‡	1101‡	-1.9 vs. -1.2 + (-1.1); -1.9 vs. -2.3; *Antagonistic action*
150	0.001 + 0.0003	NS	53.07‡	NS	*No interaction*
	0.003 + 0.001	NS	28.22‡	NS	*No interaction*
	0.01 + 0.003	NS	7.084*	4.567# [5]	-1.3 vs. -1.2 + (-1.4); -1.3 vs. -2.6; *Antagonistic action*
**Bcl-xL protein expression**					
50	0.001 + 0.0003	293.6‡	11.18*	NS	*No interaction*
	0.003 + 0.001	395.8‡	6.596*	NS	*No interaction*
	0.01 + 0.003	441.5‡	6.931*	NS	*No interaction*
100	0.001 + 0.0003	195.3‡	145.2‡	NS	*No interaction*
	0.003 + 0.001	98.07‡	99.34‡	1379‡	0 vs. -2.3 + (+1.4); 0 vs. -0.9; *Antagonistic action*
	0.01 + 0.003	NS	305.6‡	785.4‡	-1.6 vs. -2.6 + (+1.4); -1.6 vs. -1.2 [4]
150	0.001 + 0.0003	228.5‡	130.9‡	NS	*No interaction*
	0.003 + 0.001	182.0‡	167.5‡	NS	*No interaction*
	0.01 + 0.003	235.1‡	181.1‡	NS	*No interaction*
**Bax mRNA expression**					
100	0.001 + 0.0003	NS	146.0‡	14.08†	-1.6 vs. 0 + (-2.1); -1.6 vs. -2.1; *Antagonistic action*
	0.003 + 0.001	608.2‡	600.0‡	607.9‡	-1.8 vs. 0 + (-2.1); -1.8 vs. -2.1; *Antagonistic action*
	0.01 + 0.003	606.4‡	600.4‡	607.6‡	-1.5 vs. 0 + (-2.1); -1.5 vs. -2.1; *Antagonistic action*
150	0.001 + 0.0003	NS	146.6‡	NS	-1.8 vs. 0 + (-1.7); -1.8 vs. -1.7; *Potentiation*
	0.003 + 0.001	28.44‡	162.6‡	35.08‡	-1.2 vs. 0 + (-1.7); -1.6 vs. -1.7; *Antagonistic action*
	0.01 + 0.003	95.62‡	194.1‡	49.18‡	0 vs. 0 + (-1.7); 0 vs. -1.7; *Antagonistic action*
**Bax protein expression**					
50	0.001 + 0.0003	32.59‡	NS	NS	*No interaction*
	0.003 + 0.001	116.7‡	NS	NS	*No interaction*
	0.01 + 0.003	129.3‡	82.20‡	60.04‡	0 vs. +1.9 + (-1.1); 0 vs. +0.8; *Antagonistic action*
100	0.001 + 0.0003	70.24‡	198.2‡	NS	*No interaction*
	0.003 + 0.001	369.6‡	494.5‡	12.87†	0 vs. +1.6 + (-2); 0 vs. -0.4; *Antagonistic action*
	0.01 + 0.003	478.2‡	434.8‡	4.253#	0 vs. +1.9 + (-2); 0 vs. -0.1; *Antagonistic action*
150	0.001 + 0.0003	196.6‡	187.5‡	42.71‡	0 vs. +1.3 + (-5.2); 0 vs. -3.9; *Antagonistic action*
	0.003 + 0.001	205.2‡	413.5‡	NS	*No interaction*
	0.01 + 0.003	353.4‡	240.0‡	NS	*No interaction*

[1] The results of the two-way analysis of variance (ANOVA/MANOVA analysis) are presented as F values and the level of statistical significance (p). F values having $p < 0.05$ were recognized statistically significant ($^{*}p < 0.05$, $^{\dagger}p < 0.01$, $^{\ddagger}p < 0.001$, $^{\#}p = 0.06$). NS – not statistically significant. [2] To estimate the possible character of the interaction between parabens (MP + PP) and RA, the effect noted at their simultaneous treatment was compared to the sum of the effects after their separate treatment (parabens + RA effect vs. parabens effect + RA effect). Parabens effect, RA effect, and parabens + RA effect are expressed as factors of changes (-, decrease; +, increase) of a measured parameter in comparison to the control. [3] The values represent factors of changes. [4] The evaluation of the character of parabens – RA interaction was impossible. [5] Tendency to interactive impact of parabens and RA ($^{\#}p = 0.06$).

Table S9. Estimation of the main and interactive effects of parabens (methylparaben – MP and propylparaben – PP) and rosmarinic acid (RA) on the expression of cleaved caspase-3 in the human skin fibroblasts. [1,2.]

Concentration of RA (μM)	Concentrations of parabens (%) MP + PP	Parabens (MP + PP) + RA			
		Main Effect of Parabens	Main Effect of RA	Interactive Effect of Parabens – RA	Parabens + RA Effect vs. Parabens Effect + RA Effect *Possible Character of Interaction*
**Cleaved caspase-3 expression**					
50	0.001 + 0.0003	22564‡	875.8‡	874.8‡	+7.6 [3] vs. +10.8 + 0; +7.6 vs. +10.8; *Antagonistic action*
	0.003 + 0.001	55640‡	6734‡	6732‡	+9.5 vs. +18.6 + 0; +9.5 vs. +18.6; *Antagonistic action*
	0.001 + 0.003	956.4‡	NS	NS	*No interaction*
100	0.001 + 0.0003	23339‡	839.7‡	847.9‡	+7.7 vs. +10.8 + 0; +7.7 vs. +10.8; *Antagonistic action*
	0.003 + 0.001	73524‡	6775‡	5244‡	+10.7 vs. +18.6 + 0; +10.7 vs. +18.6; *Antagonistic action*
	0.001 + 0.003	41842‡	1301‡	603.2‡	+8.4 vs. +11.1 + 0; +8.4 vs. +11.1; *Antagonistic action*
150	0.001 + 0.0003	32260‡	117.1‡	121.7‡	+9.7 vs. +10.8 + 0; +9.7 vs. +10.8; *Antagonistic action*
	0.003 + 0.001	43687‡	16395‡	16448‡	+5.2 vs. +18.6 + 0; +5.2 vs. +18.6; *Antagonistic action*
	0.001 + 0.003	23634‡	4615‡	4648‡	+4.9 vs. +11.1 + 0; +4.9 vs. +11.1; *Antagonistic action*

[1] The results of the two-way analysis of variance (ANOVA/MANOVA analysis) are presented as *F* values and the level of statistical significance (*p*). *F* values having $p < 0.05$ were recognized statistically significant (*$p < 0.05$, †$p < 0.01$, ‡$p < 0.001$). NS – not statistically significant. [2] To estimate the possible character of the interaction between parabens (MP + PP) and RA, the effect noted at their simultaneous treatment was compared to the sum of the effects after their separate treatment (parabens + RA effect vs. parabens effect + RA effect). Parabens effect, RA effect, and parabens + RA effect are expressed as factors of changes (-, decrease; +, increase) of a measured parameter in comparison to the control. [3] The values represent factors of changes.

© 2020 by the authors. Licensee MDPI, Basel, Switzerland. This article is an open access article distributed under the terms and conditions of the Creative Commons Attribution (CC BY) license (http://creativecommons.org/licenses/by/4.0/).
